# Best constants in subelliptic fractional Sobolev and Gagliardo-Nirenberg inequalities and ground states on stratified Lie groups

**DOI:** 10.1007/s00526-025-03191-3

**Published:** 2025-12-13

**Authors:** Sekhar Ghosh, Vishvesh Kumar, Michael Ruzhansky

**Affiliations:** 1 Department of Mathematics, National Institute of Technology Calicut, Kozhikode, 673601 Kerala, India; 2 Department of Mathematics: Analysis, Logic and Discrete Mathematics, Ghent University, Ghent, Belgium; 3 School of Mathematical Sciences, Queen Mary University of London, London, United Kingdom

**Keywords:** 35R03, 35H20, 35P30, 22E30, 35R11

## Abstract

In this paper, we establish the sharp fractional subelliptic Sobolev inequalities and Gagliardo-Nirenberg inequalities on stratified Lie groups. The best constants are given in terms of a ground state solution of a fractional subelliptic equation involving the fractional *p*-sublaplacian ($1<p<\infty$) on stratified Lie groups. We also prove the existence of ground state (least energy) solutions to nonlinear subelliptic fractional Schrödinger equation on stratified Lie groups. Different from the proofs of analogous results in the setting of classical Sobolev spaces on Euclidean spaces given by Weinstein (Comm. Math. Phys. 87(4):576-676, 1982/1983) using the rearrangement inequality which is not available in stratified Lie groups, we apply a subelliptic version of vanishing lemma due to Lions extended in the setting of stratified Lie groups combining it with the compact embedding theorem for subelliptic fractional Sobolev spaces obtained in our previous paper (Math. Ann. 388(4):4201-4249, 2024). We also present subelliptic fractional logarithmic Sobolev inequalities with explicit constants on stratified Lie groups. The main results are new for $p=2$ even in the context of the Heisenberg group.

## Introduction and main results

The Sobolev and Gagliardo-Nirenberg inequalities play an important role in the study of partial differential equations (PDEs) (see [14, 48]). The optimal form of the classical Euclidean Sobolev inequality was independently established by Aubin [2] and Talenti [86] in 1976 through the use of symmetric rearrangement techniques. To be historically precise, the first (possible) proof of the optimal Sobolev inequality, establishing both the sharp constant and the explicit extremal functions (again via symmetric rearrangement), appears to trace back to Rodemich’s seminar note [80] from 1966. Although this reference is not widely known and remains difficult to access, we refer to [48, p. 158] for a relevant quotation and to [11] for a detailed discussion of this historical development. An alternative proof, avoiding the use of symmetric rearrangement, was later obtained through mass-transportation methods by Villani [85]. The classical Gagliardo-Nirenberg inequality in the Euclidean space $\mathbb {R}^N$ was established by Gagliardo [50] and Nirenberg [73] in their celebrated papers independently. They proved that for every $u \in W^{1,2}( \mathbb {R}^N)$, there exists a positive constant *C* such that the following inequality holds.

1.1 $$\begin{aligned} \int _{ \mathbb {R}^N}|u|^{q} dx\le C\left( \int _{ \mathbb {R}^N}|\nabla u|^{2} d x\right) ^{\frac{N(q-2)}{4}}\left( \int _{ \mathbb {R}^N}|u|^{2} d x\right) ^{\frac{2 q-N(q-2)}{4}}, \end{aligned}$$

where $2<q<2^*.$ Here, $2^*=\frac{2N}{N-2}$ for $N\ge 3$ and $2^* = +\infty$ for $N = 2$. The smallest *C* such that the inequality (1.1) holds is called the best constant and we denote it by $C_{GN, \mathbb {R}^N}$. Finding the best constants in the Gagliardo-Nirenberg inequalities has an analytical or geometrical significance and proved to be vital in studying the well-posedness of Cauchy problems and stability theorems for nonlinear parabolic PDEs. The pioneering work establishing a relationship between the best constant and least energy solutions is due to Weinstein [89]. For $N=1,$ the best constant in the Gagliardo-Nirenberg inequality was calculated by Nagy [84]. Weinstein [89] provided a form of $C_{GN, \mathbb {R}^N}$ given by the least energy solution of the following stationary Schrödinger equation.

1.2 $$\begin{aligned} -\Delta u+u=|u|^{p-2}u, \quad u \in W^{1,2}(\mathbb {R}^N). \end{aligned}$$

It is noteworthy to mention that the expression for the best constant does not depend on the least energy solution. Following these studies, there have been many contributions to the literature on the Euclidean case, which either extended and improved these inequalities or dealt with the best constants and extremisers for such inequalities. For instance, we mention [2, 4–7, 9, 10, 13, 28, 30–32, 34–37, 40, 56, 57, 69, 71, 72, 86, 90] and the references therein without making an attempt to present an exhaustive list.

In this paper, we are interested in the analysis of the best constants appearing in the fractional subelliptic Gagliardo-Nirenberg inequalities on stratified Lie groups, a subclass of nilpotent Lie groups. These groups naturally appear in analysis, representation theory, and geometry. One prominent example of such a group is the Heisenberg group. It was noted in the celebrated paper [76] by Rothschild and Stein that nilpotent Lie groups play an important role in deriving sharp subelliptic estimates for differential operators on manifolds. In 1970, Stein [83] delivered a visionary program at the ICM in Nice for studying analysis and PDEs on stratified Lie groups (see also [22]). He demonstrated it in his seminal joint paper with Rothschild on the Rothschild-Stein lifting theorem that says that a general Hörmander’s sums of squares of vector fields on manifolds can be approximated by the sublaplacian on some stratified Lie groups (see also, [43] and [77]). We also refer to Gromov [49] or Danielli, Garofalo and Nhieu [29] for general exposition from different points of view and [26, 27] for applications in mathematical models of crystal material and human vision.

The functional inequalities on Lie groups, in particular, on stratified Lie groups, have been extensively studied during the last few decades. We refer to papers [1, 20, 23, 25, 42, 51, 52, 55, 58, 61–63, 68, 79, 81], monographs [44, 78] and references therein for an excursion into the world of subelliptic functional inequalities and their applications. In this paper, we are concerned with the fractional subelliptic Sobolev and Gagliardo-Nirenberg inequalities on stratified Lie groups and their best constants. In fact, we will show, as in the seminal paper of Weinstein [89], that the best constant in the subelliptic fractional Gagliardo-Nirenberg inequality can be explicitly expressed as the least energy solution of the nonlocal subelliptic stationary Schrödinger equation on the stratified Lie group $\mathbb G$:

1.3 $$\begin{aligned} \left( -\Delta _{p, \mathbb {G}}\right) ^s u+|u|^{p-2} u = |u|^{q-2} u, \quad u \in W_{0}^{s,p}(\mathbb {G}), \end{aligned}$$

where $s \in (0, 1),$ $p \in (1, \infty )$ and $p<q<p_s^*:=\frac{Qp}{Q-ps}$ with *Q* being the homogeneous dimension of $\mathbb G$ associated with the group dilations $(D_\lambda )_{\lambda >0}$. Here, the operator $\left( -\Delta _{p, \mathbb {G}}\right) ^s$ on $\mathbb G$ is the nonlinear nonlocal counterpart of the sublaplacian on a stratified Lie group $\mathbb G.$ For $p=2,$ the operator turns out to be the fractional sublaplacian $(-\Delta _\mathbb G)^s,$ which was an object of deep investigation and its connection with different areas of mathematics in many intriguing papers [12, 45, 52, 60, 81, 82] and references cited therein. This is our guiding operator defined, initially for $u \in C_c^\infty (\mathbb G),$ by

1.4 $$\begin{aligned} (-\Delta _\mathbb G)^s(u)(x):= \lim _{\epsilon \rightarrow 0} \int _{\mathbb G\backslash B(x, \epsilon )} \frac{|u(x)-u(y)|}{|y^{-1}x|^{Q+2s}} dy = C_{Q, s}\,\, P.V. \int _{\mathbb G} \frac{|u(x)-u(y)|}{|y^{-1}x|^{Q+2s}} dy. \end{aligned}$$

It is known that for a *H*-type group the operator $(-\Delta _\mathbb G)^s,$ $s \in (0, \frac{1}{2}),$ is a multiple of the pseudo-differential operator defined as

1.5 $$\begin{aligned} \mathcal {L}_s:=2^s (-\Delta _z)^{\frac{s}{2}} \frac{\Gamma (-\frac{1}{2}\Delta _\mathbb G(-\Delta _z)^{-\frac{1}{2}}+\frac{1+s}{2}) }{\Gamma (-\frac{1}{2}\Delta _\mathbb G(-\Delta _z)^{-\frac{1}{2}}+\frac{1-s}{2})}, \end{aligned}$$

where $-\Delta _z$ is the positive Laplacian in the center of the *H*-type group $\mathbb G$ and $\Delta _\mathbb G$ is the sublaplacian on $\mathbb G.$ For more details, we refer to [12, 81, 82]. It is worth noting that $\mathcal {L}_s$ is a “conformal invariant" operator and is very important in CR geometry (see [45]). Finally, we would like to point out that the operator $(-\Delta _\mathbb G)^s$ does not coincide with the standard fractional power $-\Delta _{\mathbb G}^s$ of the sublaplacian $-\Delta _{\mathbb G}$ in the Heisenberg group or in general, *H*-type groups for any value of $s \in (0, 1),$ which is defined as

$$\begin{aligned} (- \Delta _{\mathbb G}^su)(x):=-\frac{s}{\Gamma (1-s)}\int _0^\infty \frac{1}{t^{1+s}}(H_tu(x)-u(x))\,dt, \end{aligned}$$

where $H_t:=e^{-t \Delta _\mathbb G}$ is the heat semigroup constructed by Folland [42].

On the other hand, many relevant results have been achieved during the last few years concerning the nonlinear subelliptic equations on stratified Lie groups, we cite [3, 24, 38, 39, 52–55, 66–68, 74, 75] and reference therein just to mention a few of them. Motivated by the representation (1.4) of the nonlocal operator $(-\Delta _{\mathbb G})^s,$ in this paper we will work with a more general nonlocal nonlinear operator involving the *p*-growth of the norm, known as, the fractional *p*-sublaplacian on $\mathbb G$ and defined, for $s\in (0,1)$ and $p\in [1,\infty )$, by

1.6 $$\begin{aligned} \left( -\Delta _{p,{\mathbb {G}}}\right) ^s u(x):=C_{Q,s, p}\,\, P.V. \int _{{\mathbb {G}} } \frac{|u(x)-u(y)|^{p-2}(u(x)-u(y))}{\left| y^{-1} x\right| ^{Q+p s}} dy, \quad x \in {\mathbb {G}}, \end{aligned}$$

for $u \in C_c^\infty (\mathbb G).$ To state our first result regarding the existence of the least energy solution of (1.3) involving the fractional *p*-sublaplacian on $\mathbb G$, we first recall some basic definitions. Let $\Omega \subset {\mathbb {G}}$ be an open subset. Then for $0<s<1\le p<\infty$, the fractional Sobolev space $W^{s,p}(\Omega )$ on stratified groups is defined as

1.7 $$\begin{aligned} W^{s,p}(\Omega )=\{u\in L^{p}(\Omega ): [u]_{s, p,\Omega }<\infty \}, \end{aligned}$$

endowed with the norm

1.8 $$\begin{aligned} \Vert u\Vert _{W^{s,p}(\Omega )}^p:=\Vert u\Vert _{L^p(\Omega )}^p+[u]_{s,p,\Omega }^p, \end{aligned}$$

where $[u]_{s, p,\Omega }$ denotes the Gagliardo semi-norm defined by

1.9 $$\begin{aligned} [u]_{s, p,\Omega }:=\left( \int _{\Omega } \int _{\Omega } \frac{|u(x)-u(y)|^{p}}{\left| y^{-1} x\right| ^{Q+ps}} dxdy\right) ^{\frac{1}{p}}<\infty . \end{aligned}$$

Observe that for all $u \in C_c^{\infty }(\Omega )$, we have $[u]_{s, p,\Omega }<\infty$. We define the space $W_0^{s,p}(\Omega )$ as the closure of $C_c^{\infty }(\Omega )$ with respect to the norm $\Vert u\Vert _{W^{s,p}(\Omega )}$. We would like to point out that $W_0^{s,p}(\mathbb {G})=W^{s,p}(\mathbb {G})$ (see [55]).

To state our result, we define the energy functional $I: W^{s,p}_0(\mathbb G) \rightarrow \mathbb {R}$ associated to the problem (1.3) is defined by

1.10 $$\begin{aligned} I(u):=\frac{1}{p} \iint _{\mathbb G\times \mathbb G} \frac{|u(x)-u(y)|^p}{|y^{-1} x|^{Q+ps}} dx dy +\frac{1}{p} \int _{\mathbb G} |u(x)|^{p} dx -\frac{1}{q} \int _{ \mathbb G}|u(x)|^{q} dx \end{aligned}$$

such that

$$\begin{aligned} \langle I'(u), v \rangle&= \int _{\mathbb {G}}\int _{\mathbb {G}}\frac{|u(x)-u(y)|^{p-2}(u(x)-u(y))(v(x)-v(y)}{|y^{-1}x|^{Q+ps}}dxdy \\&\quad \quad \quad + \int _{ \mathbb {G}}\left( |u|^{p-2}uv-|u|^{q-2} u v\right) dx, \end{aligned}$$

for every $$v \in W^{s,p}_0(\mathbb G).$$

Denote the Nehari manifold by

1.11 $$\begin{aligned} \mathcal {N}:=\left\{ u \in W_{0}^{s,p}(\mathbb G) \setminus \{0\}: \mathcal {L}(u)=\langle I'(u),u\rangle =0\right\} \end{aligned}$$

and set

1.12 $$\begin{aligned} d:=\inf \{I(u): u \in \mathcal {N}\} . \end{aligned}$$

We refer to Section 4 for a more detailed discussion on *I* and $\mathcal {N}.$ The following theorem is our first main result.

### Theorem 1.1

Let $\mathbb G$ be a stratified Lie group with homogeneous dimension *Q*. Let $0<s<1< p<\infty$ and $p<q<p_s^*:=\frac{Qp}{Q-ps}$. Then the problem (1.3) has a least energy solution $\phi \in W_{0}^{s,p}( \mathbb {G})$. Moreover, we have the least energy $d=I(\phi ):=\inf _{u\in \mathcal {N}}I(u)$.

This result in the classical case, that is, for (1.2), was proved by Weinstein [89], and using this, he obtained the sharp estimates of the best constant $C_{GN, \mathbb {R}^N}$ in the Gagliardo-Nirenberg inequality (1.1) by solving a minimization problem. The key ingredients of his proof were symmetric rearrangement and the compact embedding of the radial Sobolev space $W_r^{1,2}(\mathbb {R}^N)$ in $L^p(\mathbb {R}^N)$ for $N \ge 2$ and $2<q<2^*.$ It is now well-known that symmetric rearrangement techniques are not available for the stratified Lie groups and therefore one needs to seek a different proof. Chen and Rocha [25] extended the results of Weinstein [89] in the Heisenberg group by using the classical compact embedding of the Folland-Stein-Sobolev space ([51]) and Lions type vanishing lemma ([64, 65]) for the respective Folland-Stein-Sobolev space on the Heisenberg group. Based on this existence result, they ([25]) obtained the expression for the best constant of the following Gagliardo-Nirenberg inequality on the Heisenberg group:

1.13 $$\begin{aligned} \int _{\mathbb {H}^{N}}|u|^{q}dx\le C\left( \int _{\mathbb {H}^{N}}|\nabla _{H} u|^{2}dx\right) ^{\frac{Q(q-2)}{4}}\left( \int _{\mathbb {H}^{N}}|u|^{2}dx\right) ^{\frac{2q-Q(q-2)}{4}}. \end{aligned}$$

Here, $Q=2N+2$ refers to the homogeneous dimension of the Heisenberg group $\mathbb {H}^{N}$, $\nabla _{H}$ is horizontal gradient, $q\in (2, 2^*)$, where $2^*=\frac{2N}{N-2}$. The best constant in (1.13), denoted by $C_{GN, \mathbb {H}^N},$ is expressed in terms of the least energy (or ground state) solution of the subelliptic partial differential equation.

1.14 $$\begin{aligned} -\triangle _{H}u+u=|u|^{q-2}u, \;\;u\in W^{1,2}(\mathbb {H}^{N}), \end{aligned}$$

where $\triangle _{H}$ is the sublaplacian on $\mathbb {H}^{N}$, and $W^{1,2}(\mathbb {H}^{N})$ is the Sobolev space on $\mathbb {H}^{N}$ with the norm

$$\begin{aligned} \Vert u\Vert _{W^{1,2}(\mathbb {H}^{N})}:=\left( \int _{\mathbb {H}^{N}}(|\nabla _{H}u|^{2}+|u|^{2})dx\right) ^{1/2}. \end{aligned}$$

Recently, the third author and his collaborators [79] characterized the best constant of the following Gagliardo-Nirenberg inequality over a graded Lie group $\mathbb {G}$:

1.15 $$\begin{aligned} \int _{\mathbb {G}}|u(x)|^{q}dx\le C \left( \int _{\mathbb {G}}|\mathcal {R}_{1}^{\frac{a_{1}}{\nu _{1}}}u(x)|^{p}dx\right) ^{\frac{Q(q-p)-a_{2}pq}{(a_{1}-a_{2})p^{2}}} \left( \int _{\mathbb {G}}|\mathcal {R}_{2}^{\frac{a_{2}}{\nu _{2}}}u(x)|^{p}dx\right) ^{\frac{a_{1}pq-Q(q-p)}{(a_{1}-a_{2})p^{2}}} \end{aligned}$$

for all $u\in {W}^{a_1,p}(\mathbb {G})\cap {W}^{a_2,p}(\mathbb {G})$. Here $a_{1}> a_{2}\ge 0$, $1<p<\frac{Q}{a_{1}},$ $\frac{pQ}{Q-a_{2}p}\le q\le \frac{pQ}{Q-a_{1}p}$ and, $\mathcal {R}_{1}$ and $\mathcal {R}_{2}$ are positive Rockland operators of homogeneous degrees $\nu _{1}$ and $\nu _{2}$, respectively. They proved that the best constant in (1.15) can be identified by the least energy solutions of the following higher order nonlinear hypoelliptic Schrödinger equation with the power nonlinearities:

1.16 $$\begin{aligned} \mathcal {R}_{1}^{\frac{a_{1}}{\nu _{1}}}(|\mathcal {R}_{1}^{\frac{a_{1}}{\nu _{1}}}u|^{p-2}\mathcal {R}_{1}^{\frac{a_{1}}{\nu _{1}}}u)+ \mathcal {R}_{2}^{\frac{a_{2}}{\nu _{2}}}(|\mathcal {R}_{2}^{\frac{a_{2}}{\nu _{2}}}u|^{p-2}\mathcal {R}_{2}^{\frac{a_{2}}{\nu _{2}}}u)=|u|^{q-2}u. \end{aligned}$$

In this paper, we continue the aforementioned studies [25, 79] for a more general nonlocal operator, namely, the fractional *p*-sublaplacian on the stratified Lie groups. In [55], we have proved a compact embedding result for the fractional Sobolev spaces on the stratified Lie groups (see Theorem 2.4 in the next section), which is a nonlocal version of the embedding result for the Folland-Stein-Sobolev space obtained in [51]. This is one of the main tools in the proof of Theorem 1.1. The second main tool, as in the aforementioned works [25, 79], is a subelliptic version of the Lions vanishing Lemma ([64, 65]) for $W_0^{s, p}(\mathbb G),$ which is stated below and will be proved in Section 4.

### Lemma 1.2

Let $\mathbb G$ be a stratified Lie group with homogeneous dimension *Q*. Let $s \in (0, 1), p \in (1, \infty ),$ and *q* be such that $p\le q<p_s^*=\frac{Qp}{Q-ps}.$ Let $(u_k)$ be a bounded sequence in $W^{s,p}_0(\mathbb G)$ with the property

1.17 $$\begin{aligned} \liminf _{k \rightarrow \infty } \sup _{x \in \mathbb G} \int _{B(x, 1)} |u_k|^q dy =0. \end{aligned}$$

Then there exists a subsequence, also denoted by $(u_k),$ such that $u_k \rightarrow 0$ in $L^t(\mathbb G)$ for $t \in (q, p_s^*).$

We now turn our attention to deriving the fractional Gagliardo-Nirenberg inequality. We mentioned that the proof is contained in [62], but the proof is quite elementary using Hölder’s inequality, so we include it here. Recall that the following fractional subelliptic Sobolev inequality

1.18 $$\begin{aligned} \int _{\mathbb {G}}|u(x)|^{p_s^*} dx\le C\left[ \int _{\mathbb {G}}\int _{\mathbb {G}}\frac{|u(x)-u(y)|^{p}}{\left| y^{-1} x\right| ^{Q+ps}} dxdy\right] ^{\frac{p_s^*}{p}} \end{aligned}$$

for $u \in W^{s,p}_0(\mathbb G)$ was proved in [63, Theorem 2]. Now, we derive from the Hölder inequality, that

$$\begin{aligned} \int _{ \mathbb {G}}|u|^q dx=\int _{ \mathbb {G}}|u|^{ql}|u|^{q(1-l)} dx \le \left( \int _{\mathbb {G}}|u|^{p_s^*} dx\right) ^{\frac{ql}{p_s^*}}\left( \int _{ \mathbb {G}}|u|^{p} dx\right) ^{\frac{q(1-l)}{p}}, \end{aligned}$$

where $\frac{ql}{p_s^*}+\frac{q(1-l)}{p}=1$. Thus, $l=\frac{(q-p)Q}{spq}$. Therefore, eliminating *l*, we obtain

$$\begin{aligned} \int _{ \mathbb {G}}|u|^q dx\le \left( \int _{\mathbb {G}}|u|^{p_s^*} dx\right) ^{\frac{(Q-ps)(q-p)}{sp^2}}\left( \int _{ \mathbb {G}}|u|^{p} dx\right) ^{\frac{spq-Q(q-p)}{sp^2}}. \end{aligned}$$

Using the inequality (1.18), we get the following subelliptic fractional Gagliardo-Nirenberg inequality:

1.19 $$\begin{aligned} \int _{ \mathbb {G}}|u(x)|^q dx&\le C\left( \int _{\mathbb {G}}\int _{\mathbb {G}}\frac{|u(x)-u(y)|^{p}}{\left| y^{-1} x\right| ^{Q+ps}} dxdy\right) ^{\frac{Q(q-p)}{sp^2}}\left( \int _{ \mathbb {G}}|u|^{p} dx\right) ^{\frac{spq-Q(q-p)}{sp^2}}. \end{aligned}$$

The best constant for the inequality (1.19) is the smallest positive constant $C_{GN,\mathbb {G}}>0$ such that the inequality (1.19) is true. Thus, we can define $C_{GN,\mathbb {G}}$ as

1.20 $$\begin{aligned} C_{GN,\mathbb {G}}^{-1}:=\inf _{u\in W_0^{s,p}(\mathbb {G})\setminus \{0\}}\frac{\left( \int _{\mathbb {G}}\int _{\mathbb {G}}\frac{|u(x)-u(y)|^{p}}{\left| y^{-1} x\right| ^{Q+ps}} dxdy\right) ^{\frac{Q(q-p)}{sp^2}}\left( \int _{\mathbb {G}}|u(x)|^{p}dx\right) ^{\frac{spq-Q(q-p)}{sp^2}}}{\int _{\mathbb {G}}|u(x)|^{q}dx}. \end{aligned}$$

Now, we state the following theorem, which says that the best constants of the fractional Gagliardo-Nirenberg inequality can be characterized by the least energy solutions of the nonlinear subelliptic fractional Schrödinger equation (1.3).

### Theorem 1.3

Let $\mathbb G$ be a stratified Lie group with homogeneous dimension *Q*. Let $0<s<1<p<\infty$ and $Q>ps$ and $p<q<p_s^*:=\frac{Qp}{Q-ps}.$ Then the smallest positive constant $C_{GN,\mathbb {G}}$ of the Gagliardo-Nirenberg inequality (1.20) can be characterized by

1.21 $$\begin{aligned} C_{GN,\mathbb {G}}:= C_{p,q,s, Q}\, d^{\frac{p-q}{p}}, \end{aligned}$$

where $d=I(\phi ):=\inf _{u\in \mathcal {N}}I(u)$, $\phi$ being a least energy solution of (1.3) and the constant $C_{p,q,s, Q}$ is given by

1.22 $$\begin{aligned}&C_{p,q,s, Q} = \frac{pqs}{pqs-Q(q-p)}\left( \frac{Q(q-p)}{pqs-Q(p-q)}\right) ^{\frac{Q(q-p)}{sp^2}}\left( \frac{pqs-Q(q-p)}{(q-p)s} \right) ^{\frac{p-q}{p}} \end{aligned}$$

1.23 $$\begin{aligned}&= \frac{pqs}{pqs-Q(q-p)}\left( \frac{Q(q-p)}{pqs-Q(p-q)}\right) ^{\frac{Q(q-p)}{sp^2}} \left( \frac{(q-p)s}{pqs-Q(q-p)} \right) ^{\frac{p-q}{p}} \Vert \phi \Vert _{L^{p}(\mathbb {G})}^{p-q} . \end{aligned}$$

We would like to mention that Theorem 1.3 provides a characterization of the optimal constant $C_{GN,\mathbb {G}}$ in the inequality (1.19), but not an explicit expression for it, since the value of $C_{GN,\mathbb {G}}$ depends on the quantity $d$. However, in certain cases, for instance, when $p = 2$ and $\mathbb {G} = \mathbb {R}^N$ or $\mathbb {G} = \mathbb {H}^N$ (the Heisenberg group), one can compute the value of $d$ or obtain an estimate of it, which in turn yields an estimate of $C_{GN,\mathbb {G}}$. For general $p$, obtaining such an estimate of $d$ appears to be out of reach, even in the Euclidean setting, to the best of our knowledge. To illustrate it for $p=2$ and $\mathbb G=\mathbb {H}^N$ with homogeneous dimension $Q=2N+2,$ we recall (see [87, Theorem 1.2 and Theorem 3.1] and [19]) that the best constant in (1.18) is given by

$$S_{Opt, N,s, \mathbb {H}^N} := \left( \frac{ 2^{-N-2+s}\,\pi ^{-N-1}\, \Gamma ^2\left( \frac{N+1-s}{2}\right) }{ 4^{\,s\frac{2N+1}{N+1}}\, \pi ^{\,s\frac{2N+1}{2N+2}}\, |\Gamma (-s)|\, \left( \frac{\Gamma \left( N+\tfrac{1}{2}\right) }{\Gamma (2N+1)}\right) ^{\frac{s}{N+1}} } \right) ^{\!\frac{N+1}{N-1+s}}.$$

Then, it is clear from Theorem 1.3 along with (1.18) and (1.19) that

$$\begin{aligned} C_{GN, \mathbb {H}^N}:= C_{p,q,s, 2N+2}\,\, d^{\frac{p-q}{p}} \le S_{Opt, N,s, \mathbb {H}^N}, \end{aligned}$$

which further gives an estimate of *d* given by

$$\begin{aligned} 0<d \le \left( \frac{S_{Opt, N,s, \mathbb {H}^N}}{C_{p,q,s, 2N+2}}\right) ^{\frac{p}{p-q}}. \end{aligned}$$

This provides an explicit constant in (1.19) in the case $p = 2$ for the Heisenberg group. In the local Euclidean setting, the Aubin–Talenti profiles, which characterize the least energy solutions, are explicit and help to determine the best constant in the $p$-Gagliardo–Nirenberg inequalities; we refer to [11, 30] for more details. For the Euclidean fractional Sobolev inequality (that is, (1.18) for $p = 2$ and $\mathbb {G} = \mathbb {R}^N$), the profile of the optimizers is explicitly known, and hence the best constant in this setting can be determined (see [21]). Using this, one can obtain an estimate for the best constant in (1.19) for $p = 2$ and $\mathbb {G} = \mathbb {R}^N$; however, to the best of our knowledge, no explicit expression of the optimal constant is known (see [9, 57]). The best constants and optimizers for subelliptic (fractional) Sobolev inequality result for $\mathbb G=\mathbb {H}^N$ are obtained in [46, 58, 59, 87]. Unfortunately, in the nonlocal and nonlinear case $(p \ne 2)$ for $\mathbb {R}^N$, the classification of the optimizers for (1.18) is a very difficult and still open problem, mainly due to the lack of a suitable Kelvin transform and an equivalent integral representation. However, the decay rate at infinity of the optimizers is known; we refer to [16] and the references therein for further details. We also refer to [52] for similar results concerning the decay rate at infinity of the optimizers of (1.18) with $p = 2$ on stratified Lie groups.

We also obtain a similar result for the fractional subelliptic Sobolev inequality on stratified Lie groups. For this, we recall the following Sobolev inequality from [55, Theorem 1.1]

1.24 $$\begin{aligned} \left( \int _{\mathbb {G}}|u(x)|^{q}dx\right) ^{\frac{p}{q}}\le C\left[ \int _{\mathbb {G}}\int _{\mathbb {G}}\frac{|u(x)-u(y)|^{p}}{\left| y^{-1} x\right| ^{Q+ps}} dxdy+\int _{\mathbb {G}}|u(x)|^{p}dx\right] , \end{aligned}$$

for $u \in W^{s,p}_0(\mathbb G).$ Let us define the best (optimal) constant $C_{S, \mathbb G}>0$ in (1.24) as the least positive constant for which the inequality (1.24) holds, namely,

1.25 $$\begin{aligned} C_{S, \mathbb {G}}^{-1}:=\inf _{u\in W_0^{s,p}(\mathbb {G})\setminus \{0\}}\frac{\int _{\mathbb {G}}\int _{\mathbb {G}}\frac{|u(x)-u(y)|^{p}}{\left| y^{-1} x\right| ^{Q+ps}} dxdy+\int _{\mathbb {G}}|u(x)|^{p}dx}{\left( \int _{\mathbb {G}}|u(x)|^{q}dx\right) ^{\frac{p}{q}}}. \end{aligned}$$

The following result expresses the best constant of the fractional Sobolev inequality (1.24) in terms of the least energy solution of (1.3).

### Theorem 1.4

Let $\mathbb G$ be a stratified Lie group with homogeneous dimension *Q*. Let $0<s<1<p<\infty$ and $Q>ps$ and $p<q<p_s^*:=\frac{Qp}{Q-ps}.$ Let $C_{S, \mathbb {G}}$ be the least positive constant such that the inequality (1.24) holds. Then we have

1.26 $$\begin{aligned} C_{S,\mathbb {G}}&=\left( \frac{pq}{q-p} \right) ^{\frac{p-q}{q}} d^{\frac{p-q}{q}}, \end{aligned}$$

1.27 $$\begin{aligned}&=\left( \frac{spq}{spq-Q(q-p)}\right) ^{\frac{p-q}{q}} \Vert \phi \Vert _{L^p(\mathbb G)}^{\frac{p(p-q)}{q}} \end{aligned}$$

where $d=I(\phi ):=\inf _{u\in \mathcal {N}}I(u)$, $\phi$ being a least energy solution of (1.3).

### Remark 1.5

We do not know if $\phi$ is a unique least energy solution of (1.3). But equality in (1.26) and (1.21) implies that $C_{S,\mathbb {G}}$ and $C_{GN,\mathbb {G}}$ are independent of the choice of $\phi$. Further, we want to mention here that if we replace *Q* with the Euclidean dimension *N*, and $\mathbb {G}$ by $\mathbb {R}^N$, then both of these constants reduce to the classical results. For instance, for $\mathbb {G}=\mathbb {R}^N$, $s=1$, $p=2,$ it is the same as the result due to Weinstein [89] whereas for $\mathbb {G}=\mathbb {H}^N$, $s=1$, $p=2,$ it coincides with the result of Chen and Rocha [25]

### Remark 1.6

We can easily establish a relation between the sharp constant $C_{GN, \mathbb G}$ in the fractional subelliptic Gagliardo-Nirenberg inequality (1.20) and the sharp constant $C_{S, \mathbb G}$ in the fractional subelliptic Sobolev inequality (1.24) on stratified Lie groups. Indeed, from Theorem 1.3 we have

$$\begin{aligned} C_{GN,\mathbb {G}}&=\frac{pqs}{pqs-Q(q-p)} \left( \frac{Q(q-p)}{pqs-Q(q-p)}\right) ^{\frac{Q(p-q)}{sp^2}}\left( \frac{pqs-Q(q-p)}{(q-p)s} d\right) ^{\frac{p-q}{p}}, \end{aligned}$$

which implies that

$$\begin{aligned} C^{\frac{p}{q}}_{GN, \mathbb G}=\frac{pqs}{pqs-Q(q-p)} \left( \frac{Q(q-p)}{pqs-Q(q-p)}\right) ^{\frac{Q(p-q)}{pqs}} C_{S, \mathbb G}. \end{aligned}$$

Now, we derive the logarithmic fractional Sobolev inequalities on stratified Lie groups with an explicit constant with the help of Theorem 1.3 and Theorem 1.4. It is worth emphasizing that these results are also new in the Euclidean framework. For the results on *p*-logarithmic Sobolev inequalities with optimal constants in the Euclidean space, we refer to [33, 47] and the references therein. In this regard, our result is the following theorem.

### Theorem 1.7

Let $\mathbb G$ be a stratified Lie group with homogeneous dimension *Q*. Let $s \in (0, 1),$ $p \in (1, \infty ), Q>ps$ and $p<q<p^*:=\frac{Qp}{Q-ps}.$ Then for any $u \ne 0,$ we have

1.28 $$\begin{aligned}&\int _{\mathbb G} \frac{|u(x)|^p}{\Vert u\Vert _{L^p(\mathbb G)}^p} \log \left( \frac{|u(x)|^p}{\Vert u\Vert ^p_{L^p(\mathbb G)}} \right) dx \nonumber \\  &\quad \le \frac{Q}{s} \log \left( C_{S, \mathbb G, p}\frac{\left[ \int _{\mathbb {G}}\int _{\mathbb {G}}\frac{|u(x)-u(y)|^{p}}{\left| y^{-1} x\right| ^{Q+ps}} dxdy+\int _{\mathbb {G}}|u(x)|^{p})dx\right] }{\Vert u\Vert _{L^p(\mathbb G)}^p} \right) , \end{aligned}$$

where $C_{S, \mathbb G, p}$ is given by

$$\begin{aligned} C_{S, \mathbb G, p}:=\left( \frac{s}{Qd}\right) ^{\frac{sp}{Q}}. \end{aligned}$$

Here $d=I(\phi ):=\inf _{u\in \mathcal {N}}I(u)$, $\phi$ being a least energy solution of (1.3).

Before stating our next result, let us discuss the idea of the proof of Theorem 1.7 and the next theorem. The method is quite simple and uses the logarithmic Hölder inequalities (see Theorem 7.2) combined with the subelliptic fractional Gagliardo-Nirenberg and Sobolev inequalities. For the Euclidean case, this method was possibly first used by Merker [70] and later in many papers in several different scenarios, for example, [57] for the logarithmic Sobolev and Gagliardo-Nirenberg inequalities under the Lorentz norms and [17, 18, 61] for logarithmic Sobolev and Gagliardo-Nirenberg inequalities on Lie groups. We now state our last result concerning a different version of the logarithmic fractional Sobolev inequality with a homogeneous norm. This result was first proved in [62] without an explicit constant.

### Theorem 1.8

Let $\mathbb G$ be a stratified Lie group with homogeneous dimension *Q*. Let $s \in (0, 1),$ $p \in (1, \infty ), Q>ps$ and $p<q<p^*:=\frac{Qp}{Q-ps}.$ Then for any $u \ne 0,$ we have

1.29 $$\begin{aligned} \int _{\mathbb G} \frac{|u(x)|^p}{\Vert u\Vert _{L^p(\mathbb G)}^p} \log \left( \frac{|u(x)|^p}{\Vert u\Vert ^p_{L^p(\mathbb G)}} \right) dx \le \frac{Q}{s} \log \left( C_{GN, \mathbb G}^{\frac{ps}{Q(q-p)}}\frac{\left( \int _{\mathbb {G}}\int _{\mathbb {G}}\frac{|u(x)-u(y)|^{p}}{\left| y^{-1} x\right| ^{Q+ps}} dxdy\right) ^{\frac{1}{p}}}{\Vert u\Vert _{L^p(G)}} \right) , \end{aligned}$$

where $C_{GN, \mathbb G}$ is the best constant in the fractional subelliptic Gagliardo-Nirenberg inequality given by

$$\begin{aligned} C_{GN, \mathbb G}:=\frac{pqs}{pqs-Q(q-p)}\left( \frac{Q(q-p)}{pqs-Q(q-p)}\right) ^{\frac{Q(p-q)}{sp^2}}\left( \frac{pqs-Q(q-p)}{(q-p)s} d\right) ^{\frac{p-q}{p}}. \end{aligned}$$

Here $d=I(\phi ):=\inf _{u\in \mathcal {N}}I(u)$, $\phi$ being a least energy solution of (1.3).

The rest of the paper is organized as follows: in Section 2 we present some basics of analysis on stratified Lie groups and fractional subelliptic Sobolev spaces. Section 3 will be devoted to the proof of the Lions vanishing lemma for stratified Lie groups. The proof of Theorem 1.1 is contained in Section 4. The proof of Theorem 1.3 regarding the best constant of the fractional Gagliardo-Nirenberg inequality on stratified Lie groups is given in Section 5. The proof of Theorem 1.4 is presented in Section 6. We end this paper in Section 7 by discussing the proof of Theorem 1.7 and Theorem 1.8, talking about the logarithmic version of fractional subelliptic Sobolev inequalities with explicit constants.

## Preliminaries: Stratified Lie groups and fractional Sobolev spaces

Prior to proceeding with the main results, we recall some preliminary tools of the stratified Lie groups and the fractional Sobolev spaces defined on them. There are many ways to introduce the notion of stratified Lie groups, for instance, one may refer to books and monographs [8, 41, 44, 78]. In his seminal paper [42], Folland extensively investigated the properties of function spaces on these groups. For precise studies and properties on stratified Lie groups, we refer [8, 41, 42, 44, 51, 55].

### Definition 2.1

A Lie group $\mathbb {G}$ (on $\mathbb {R}^N$) is said to be homogeneous if, for each $\lambda >0$, there exists an automorphism $D_{\lambda }:\mathbb {G} \rightarrow \mathbb {G}$ defined by $D_{\lambda }(x)=(\lambda ^{r_1}x_1, \lambda ^{r_2}x_2,..., \lambda ^{r_N}x_N)$ for $r_i>0,\,\forall \, i=1,2,...,N$. The map $D_\lambda$ is called a *dilation* on $\mathbb {G}$.

For simplicity, we sometimes prefer to use the notation $\lambda x$ to denote the dilation $D_{\lambda }x$. Note that, if $\lambda x$ is a dilation, then $\lambda ^r x$ is also a dilation. The number $Q=r_1+r_2+...+r_N$ is called the homogeneous dimension of the homogeneous Lie group $\mathbb {G}$, and the natural number *N* represents the topological dimension of $\mathbb {G}.$ The Haar measure on $\mathbb {G}$ is denoted by *dx* and it is nothing but the usual Lebesgue measure on $\mathbb {R}^N.$

### Definition 2.2

A homogeneous Lie group $\mathbb {G} = (\mathbb {R}^N, \circ )$ is called a stratified Lie group (or a homogeneous Carnot group) if the following two conditions are fulfilled:

(i) For some natural numbers $N_1 + N_2+... + N_k = N$ the decomposition $\mathbb {R}^N = \mathbb {R}^{N_1} \times \mathbb {R}^{N_2} \times ... \times \mathbb {R}^{N_k}$ holds, and for each $\lambda > 0$ there exists a dilation of the form $D_{\lambda }(x)= (\lambda x^{(1)}, \lambda ^2 x^{(2)},..., \lambda ^{k}x^{(k)})$ which is an automorphism of the group $\mathbb {G}$. Here $x^{(i)}\in \mathbb {R}^{N_i}$ for $i = 1,2,..., k$.
(ii) With $N_1$ as in the above decomposition of $\mathbb {R}^N$, let $X_1,..., X_{N_1}$ be the left invariant vector fields on $\mathbb {G}$ such that $X_k(0) = \frac{\partial }{\partial x_k}|_0$ for $k = 1,..., N_1$. Then the Hörmander condition $rank(Lie\{X_1,..., X_{N_1} \}) = N$ holds for every $x \in \mathbb {R}^{N}$. In other words, the Lie algebra corresponding to the Lie group $\mathbb {G}$ is spanned by the iterated commutators of $X_1,..., X_{N_1}$.

Here *k* is called the step of the stratified Lie group. Note that, in this case, the homogeneous dimension becomes $Q=\sum _{i=1}^{i=k}iN_i$. Furthermore, the left-invariant vector fields $X_j$ satisfy the divergence theorem, and they can be written explicitly as

2.1 $$\begin{aligned} X_i=\frac{\partial }{\partial x_i^{(1)}} + \sum _{j=2}^{k}\sum _{l=1}^{N_1} a^{(j)}_{i,l}(x^1, x^2, ..., x^{j-1})\frac{\partial }{\partial x_l^{(j)}}. \end{aligned}$$

For simplicity, we set $n=N_1$ in the above Definition 2.2.

An absolutely continuous curve $\gamma :[0,1]\rightarrow \mathbb {R}$ is said to be admissible, if there exist functions $c_i:[0,1]:\rightarrow \mathbb {R}$, for $i=1,2,...,n,$ such that

$$\begin{aligned} {\dot{\gamma }(t)}=\sum _{i=1}^{i=n}c_i(t)X_i(\gamma (t))~\text {and}~ \sum _{i=1}^{i=n}c_i(t)^2\le 1. \end{aligned}$$

Observe that the functions $c_i$ may not be unique as the vector fields $X_i$ may not be linearly independent. For any $x,y\in \mathbb {G}$ the Carnot-Carathéodory distance is defined as

$$ \begin{aligned}\rho _{cc}(x,y)=\inf \{l>0: ~\text {there exists an admissible}~ \gamma :[0,l]\rightarrow \mathbb {G} ~\text {with}~ \gamma (0)=x ~{ \& }~ \gamma (l)=y \}.\end{aligned}$$

The Hörmander condition for the vector fields $X_1,X_2,...X_{N_1}$ ensures that $\rho _{cc}$ is a metric. The space $(\mathbb {G}, \rho _{cc})$ is is known as a Carnot-Carathéodory space.

Let us now define the quasi-norm on the stratified Lie group $\mathbb {G}$.

### Definition 2.3

A continuous function $|\cdot |: \mathbb {G} \rightarrow \mathbb {R}^{+}$ is said to be a homogeneous quasi-norm on a stratified Lie group $\mathbb {G}$ if it satisfies the following conditions:

(i) (definiteness): $|x| = 0$ if and only if $x = 0$.
(ii) (symmetric): $|x^{-1}| = |x|$ for all $x \in \mathbb {G}$, and
(iii) (1-homogeneous): $|\lambda x| = \lambda |x|$ for all $x \in \mathbb {G}$ and $\lambda >0$.

An example of a quasi-norm on $\mathbb {G}$ is the norm defined as $d(x):=\rho _{cc}(x, 0),\,\, x\in \mathbb {G}$, where $\rho$ is the Carnot-Carathéodory distance related to Hörmander vector fields on $\mathbb {G}.$ It is known that all homogeneous quasi-norms are equivalent on $\mathbb {G}.$ In this paper, we will work with a left-invariant homogeneous distance $d(x, y):=|y^{-1} \circ x|$ for all $x, y \in \mathbb {G}$ induced by the homogeneous quasi-norm of $\mathbb {G}.$

The sublaplacian (or Horizontal Laplacian) on $\mathbb {G}$ is defined as

2.2 $$\begin{aligned} \mathcal {L}:=X_{1}^{2}+\cdots +X_{N_1}^{2}. \end{aligned}$$

The horizontal gradient on $\mathbb {G}$ is defined as

2.3 $$\begin{aligned} \nabla _{\mathbb {G}}:=\left( X_{1}, X_{2}, \cdots , X_{N_1}\right) . \end{aligned}$$

The horizontal divergence on $\mathbb {G}$ is defined by

2.4 $$\begin{aligned} \operatorname {div}_{\mathbb {G}} v:=\nabla _{\mathbb {G}} \cdot v. \end{aligned}$$

For $p \in (1,+\infty )$, we define the *p*-sublaplacian on the stratified Lie group $\mathbb {G}$ as

2.5 $$\begin{aligned} \Delta _{\mathbb {G},p} u:=\operatorname {div}_{\mathbb {G}}\left( \left| \nabla _{\mathbb {G}} u\right| ^{p-2} \nabla _{\mathbb {G}} u\right) . \end{aligned}$$

Let $\Omega$ be a Haar measurable subset of $\mathbb {G}$. Then $\mu (D_{\lambda }(\Omega ))=\lambda ^{Q}\mu (\Omega )$ where $\mu (\Omega )$ is the Haar measure of $\Omega$. The quasi-ball of radius *r* centered at $x\in \mathbb {G}$ with respect to the quasi-norm $|\cdot |$ is defined as

2.6 $$\begin{aligned} B(x, r)=\left\{ y \in \mathbb {G}: \left| y^{-1} \circ x\right| <r\right\} . \end{aligned}$$

Observe that *B*(*x*, *r*) can be obtained by the left translation by *x* of the ball *B*(0, *r*). Furthermore, *B*(0, *r*) is the image under the dilation $D_{r}$ of *B*(0, 1). Thus, we have $\mu (B(x,r))=r^Q\mu (B(0,1))$ for all $x\in \mathbb {G}$.

The simplest example of a stratified Lie group is the Heisenberg group $\mathbb {H}^N$ with the underlying manifold $\mathbb {R}^{2N+1}:=\mathbb {R}^N\times \mathbb {R}^N\times \mathbb {R}$ for $N\in \mathbb {N}$. For $(x, y, t),(x', y', t')\in \mathbb {H}^N$ the multiplication in $\mathbb {H}^N$ is given by

$$\begin{aligned} (x, y, t) \circ (x', y', t')=(x+x', y+y', t+t'+2 (\langle x',y\rangle )-\langle x,y'\rangle ), \end{aligned}$$

where $(x, y, t), (x', y', t') \in \mathbb {R}^{N}\times \mathbb {R}^{N}\times \mathbb {R}$ and $\langle \cdot ,\cdot \rangle$ represents the inner product on $\mathbb {R}^N$. The homogeneous structure of the Heisenberg group $\mathbb {H}^N$ is provided by the following dilation, for $\lambda >0,$

$$\begin{aligned} D_{\lambda }(x,y,t)=(\lambda x, \lambda y, \lambda ^2 t). \end{aligned}$$

The homogeneous dimension *Q* of $\mathbb {H}^N$ is given by $2N+2:= N+N+2$ while the topological dimension of $\mathbb {H}^N$ is $2N+1.$ A homogeneous norm for $\mathbb {H}^N$ is given by

$$\begin{aligned} |(x, y, t)|=\left( \sum _{i=1}^{n}(x_{i}^{2}+y_{i}^{2})^{2}+16t^{2}\right) ^{\frac{1}{4}}, \end{aligned}$$

for $(x,y,t) \in \mathbb {H}^{n}$. We define the homogeneous norm The left-invariant vector fields $\{X_i, Y_i\}_{i=1}^N$ defined below form a basis for the Lie algebra corresponding to the Heisenberg group $\mathbb {H}^N$:

2.7 $$\begin{aligned} X_i&=\frac{\partial }{\partial x_i}+2y_i\frac{\partial }{\partial t}; Y_i=\frac{\partial }{\partial y_i}-2x_i\frac{\partial }{\partial t}~\text {and}~ T=\frac{\partial }{\partial t}, ~\text {for}~i=1, 2,..., N. \end{aligned}$$

It is easy to see that $[X_i,Y_i]=-4T$ for $i=1,2,...,N$ and

$$\begin{aligned} [X_i,X_j]=[Y_i,Y_j]=[X_i,Y_j]=[X_i,T]=[Y_j,T]=0 \end{aligned}$$

for all $i\ne j$ and these vector fields satisfy the Hörmander rank condition. Consequently, the sub-Laplacian on $\mathbb {H}^N$ is given by

$$\begin{aligned} \Delta _{\mathbb {H}^N}:=\sum _{i=1}^N (X_i^2+Y_i^2). \end{aligned}$$

We are now in a position to define the notion of fractional Sobolev-Folland-Stein type spaces related to our study.

Let $\Omega \subset {\mathbb {G}}$ be an open subset. Then for $0<s<1< p<\infty$, the fractional Sobolev space $W^{s,p}(\Omega )$ on stratified groups is defined as

2.8 $$\begin{aligned} W^{s,p}(\Omega )=\{u\in L^{p}(\Omega ): [u]_{s, p,\Omega }<\infty \}, \end{aligned}$$

endowed with the norm

2.9 $$\begin{aligned} \Vert u\Vert _{W^{s,p}(\Omega )}^p=\Vert u\Vert _{L^p(\Omega )}^p+[u]_{s,p,\Omega }^p, \end{aligned}$$

where $[u]_{s, p,\Omega }$ denotes the Gagliardo semi-norm defined by

2.10 $$\begin{aligned} [u]_{s, p,\Omega }:=\left( \int _{\Omega } \int _{\Omega } \frac{|u(x)-u(y)|^{p}}{\left| y^{-1} x\right| ^{Q+ps}} dxdy\right) ^{\frac{1}{p}}<\infty . \end{aligned}$$

Observe that for all $\phi \in C_c^{\infty }(\Omega )$, we have $[u]_{s, p,\Omega }<\infty$. We define the space $W_0^{s,p}(\Omega )$ as the closure of $C_c^{\infty }(\Omega )$ with respect to the norm $\Vert u\Vert _{W^{s,p}(\Omega )}$. We would like to point out that $W_0^{s,p}(\mathbb {G})=W^{s,p}(\mathbb {G})$ (see [55]).

Now for an open bounded subset $\Omega \subset {\mathbb {G}}$, define the space $X_0^{s,p}(\Omega )$ as the closure of $C_c^{\infty }(\Omega )$ with respect to the norm $\Vert u\Vert _{L^p(\Omega )}+[u]_{s, p,\mathbb {G}}$. Note that the spaces $X_0^{s,p}(\Omega )$ and $W_0^{s,p}(\Omega )$ are different even in the Euclidean case unless $\Omega$ is an extension domain (see [72]). Note that when $\Omega =\mathbb {G}$, then we have $X_0^{s,p}(\mathbb {G})=W_0^{s,p}(\mathbb {G})=W^{s,p}(\mathbb {G})$. The space $X_0^{s,p}(\Omega )$ is a reflexive Banach space for $1< p<\infty$.

The space $X_0^{s,p}(\Omega )$ can be defined as a closure of $C_c^{\infty }(\Omega )$ with respect to the homogeneous norm $[u]_{s,p, \mathbb {G}}$. That is

$$\begin{aligned} \Vert u\Vert _{X_0^{s,p}(\Omega )}\cong [u]_{s, p,\mathbb {G}}~\text {for all}~u\in X_0^{s,p}(\Omega ). \end{aligned}$$

Moreover, the construction of the space $X_0^{s,p}(\Omega )$ suggests that if $\Omega$ is bounded then we can represent $X_0^{s,p}(\Omega )$ as follows:

$$\begin{aligned} X_0^{s,p}(\Omega )=\{u\in W^{s, p}({\mathbb {G}}): u=0~\text {in}~{\mathbb {G}}\setminus \Omega \}. \end{aligned}$$

For $$u\in X_0^{s,p}(\Omega )$$,

$$\begin{aligned} [u]_{s, p,\mathbb {G}}^p&=\iint _{\mathbb {G} \times \mathbb {G}} \frac{|u(x)-u(y)|^{p}}{\left| y^{-1} x\right| ^{Q+p s}} dxdy=\iint _{\mathbb {G} \times \mathbb {G}\setminus (\Omega ^c\times \Omega ^c)} \frac{|u(x)-u(y)|^{p}}{\left| y^{-1} x\right| ^{Q+p s}} dxdy. \end{aligned}$$

We conclude this section with the following two definitions. For $s\in (0,1)$ and $p\in (1,\infty )$, we define the fractional *p*-sublaplacian as

2.11 $$\begin{aligned} \left( -\Delta _{p,{\mathbb {G}}}\right) ^s u(x):=C_{Q,s, p}\,\, P.V. \int _{{\mathbb {G}} } \frac{|u(x)-u(y)|^{p-2}(u(x)-u(y))}{\left| y^{-1} x\right| ^{Q+p s}} dy, \quad x \in {\mathbb {G}}. \end{aligned}$$

For any $\varphi \in X_0^{s,p}(\Omega )$, we have

2.12 $$\begin{aligned} \langle \left( -\Delta _{p,{\mathbb {G}}}\right) ^s u,\varphi \rangle = \iint _{\mathbb {G} \times \mathbb {G}} \frac{|u(x)-u(y)|^{p-2}(u(x)-u(y))(\varphi (x)-\varphi (y))}{\left| y^{-1} x\right| ^{Q+p s}} dxdy. \end{aligned}$$

Let us recall the following theorem on the subelliptic fractional embedding results proved in [55].

### Theorem 2.4

[55, Theorem 1.1] Let $\mathbb {G}$ be a stratified Lie group of homogeneous dimension *Q*, and let $\Omega \subset \mathbb {G}$ be an open set. Let $0<s<1\le p<\infty$ and $Q>sp.$ Then the fractional Sobolev space $X_0^{s, p}(\Omega )$ is continuously embedded in $L^r(\Omega )$ for $p\le r\le p_s^*:=\frac{Qp}{Q-sp}$, that is, there exists a positive constant $C=C(Q,s,p, \Omega )$ such that for all $u\in X_0^{s, p}(\Omega )$, we have

2.13 $$\begin{aligned} \Vert u\Vert _{L^r(\Omega )}\le C \Vert u\Vert _{X_0^{s,p}(\Omega )} \end{aligned}$$

In particular, we have

2.14 $$\begin{aligned} \Vert u\Vert _{L^r(\mathbb {G})}\le C_{Q, s, p} \Vert u\Vert _{W^{s,p}(\mathbb {G})}. \end{aligned}$$

Moreover, if $\Omega$ is bounded, then the following embedding

2.15 $$\begin{aligned} X_0^{s,p}(\Omega ) \hookrightarrow L^r(\Omega ) \end{aligned}$$

is continuous for all $r\in [1,p_s^*]$ and is compact for all $r\in [1,p_s^*)$.

## A subelliptic version of Lions vanishing lemma for $W_0^{s,p}(\mathbb {G})$

In this section, we prove the subelliptic vanishing lemma established for classical Sobolev space by Lions [64, 65] in his seminal papers. Recently, this lemma was obtained by Wang [88] in the setting of the Heisenberg group. We follow the proof presented in a recent paper [90] dealing with the Euclidean space scenario.

### Lemma 3.1

Let $\mathbb G$ be a stratified Lie group with homogeneous dimension *Q*. Let $s \in (0, 1), p \in (1, \infty ),$ and let *q* be such that $p\le q<p_s^*=\frac{Qp}{Q-ps}.$ Let $(u_k)$ be a bounded sequence in $W^{s,p}_0(\mathbb G)$ with the property

3.1 $$\begin{aligned} \liminf _{k \rightarrow \infty } \sup _{x \in \mathbb G} \int _{B(x, 1)} |u_k|^q dy =0. \end{aligned}$$

Then there exists a subsequence, again denoted by $(u_k),$ such that $u_k \rightarrow 0$ in $L^r(\mathbb G)$ for $r \in (q, p_s^*).$

### Proof

Let $(u_k)$ be a sequence in $W^{s, p}_0(\mathbb G)$ and $q \in [p, p_s^*).$ We will first prove that

3.2 $$\begin{aligned} \int _{\mathbb G} |u_k|^r dy \le C \Big ( \sup _{x \in \mathbb G} \int _{B(x, 1)} |u_k|^q dy \Big )^{\frac{p_s^*-r}{p_s^*-q}} \Vert u_k\Vert ^p_{W^{s, p}_0(\mathbb G)} \end{aligned}$$

for any $r \in (q, p_s^*)$ and for all $u_k \in W^{s, p}_0(\mathbb G).$

For any $r \in (q, p_s^*)$ we write $\frac{1}{r}=\frac{\beta }{q}+\frac{1-\beta }{p_s^*}$ for some $\beta \in (0, 1).$ Then, using the Hölder inequality we have

$$\begin{aligned} \Vert u_k\Vert _{L^r(B(x, 1))}^r \le \Vert u_k\Vert _{L^q(B(x, 1))}^{q \beta } \Vert u_k\Vert _{L^{p_s^*}(B(x, 1))}^{(1-\beta )p_s^* }. \end{aligned}$$

Now, by choosing $\beta = \frac{p_s^*-r}{p_s^*-q} \in (0, 1)$ and using the local Sobolev embedding (2.13) we get

$$\begin{aligned} \int _{B(x, 1)} |u_k|^r dy&\le \Bigg ( \int _{B(x, 1)} |u_k|^q dy \Bigg )^{\frac{p_s^*-r}{p_s^*-q}} \Bigg ( \int _{B(x, 1)} |u_k|^{p_s^*} dy \Bigg )^{\frac{r-q}{p_s^*-q}} \\&\le C \Bigg ( \int _{B(x, 1)} |u_k|^q dy \Bigg )^{\frac{p_s^*-r}{p_s^*-q}} \Vert u_k\Vert _{X^{s, p}_0(B(x, 1))}^{p_s^*\frac{r-q}{p_s^*-q}}. \end{aligned}$$

Next, for this choice of $\beta$ we have $r = \frac{p(p_s^*-q)+p_s^*q}{p_s^*}$ so that $\frac{p_s^*}{p}\frac{r-q}{p_s^*-q}=p.$ Thus we get

3.3 $$\begin{aligned} \int _{B(x, 1)} |u_k|^r dy \le C \Bigg ( \int _{B(x, 1)} |u_k|^q dy \Bigg )^{\frac{p_s^*-r}{p_s^*-q}} \Vert u_k\Vert _{X^{s, p}_0(B(x, 1))}^{p}. \end{aligned}$$

Using the covering lemma [41, Lemma 5.7.5], we can choose a covering $\{B(x_i, 1)\}_{i=1}^\infty$ of $\mathbb G$ such that no point of $\mathbb G$ belongs to more than $(4C_0^2)^Q$ such balls, where $C_0$ is the constant appearing in the triangle inequality of the quasi-norm $|\cdot |$ on $\mathbb G$.

Therefore, we have

3.4 $$\begin{aligned} \int _{\mathbb G} |u_k|^r dy&\le \int _{\cup _{i} B(x_i, 1)} |u_k|^r dy \nonumber \le \sum _{i} \int _{B(x_i, 1)} |u_k|^r dy \nonumber \\&\le C \sum _{i} \Bigg ( \int _{B(x_i, 1)} |u_k|^q dy \Bigg )^{\frac{p_s^*-r}{p_s^*-q}} \Vert u_k\Vert _{X^{s, p}_0(B(x_i, 1))}^{p} \nonumber \\&\le C (4C_0)^Q \Bigg ( \sup _{x \in \mathbb G} \int _{B(x, 1)} |u_k|^q dy \Bigg )^{\frac{p_s^*-r}{p_s^*-q}} \sum _{i} \Vert u_k\Vert _{X^{s, p}_0(B(x_i, 1))}^{p} \nonumber \\&\le C (4C_0)^Q\Big ( \sup _{x \in \mathbb G} \int _{B(x, 1)} |u_k|^q dy\Big )^{\frac{p_s^*-r}{p_s^*-q}} \Vert u_k\Vert ^p_{W^{s, p}_0(\mathbb G)}, \end{aligned}$$

proving (3.2). Thus, applying the hypothesis (3.1) in (3.2) we conclude that

$$\begin{aligned} \liminf _{k \rightarrow \infty } \int _{\mathbb G} |u_k|^rdy =0, \end{aligned}$$

for $r \in (q, p_s^*).$ This implies that $u_k \rightarrow 0$ in $L^r(\mathbb G)$ for $r \in (q, p_s^*).$ $\square$

## Existence of least energy solutions for (1.3)

This section is devoted to proving Theorem 1.1. Throughout this section, we always assume that $p<q<p_s^*$. For the convenience of readers, let us recall the subelliptic fractional stationary Schrödinger equation (1.3) which is the main object of study in Theorem 1.1:

4.1 $$\begin{aligned} \left( -\Delta _{p, \mathbb {G}}\right) ^s u+|u|^{p-2} u = |u|^{q-2} u, \quad u \in W_{0}^{s,p}(\mathbb {G}), \end{aligned}$$

where $p<q<p_s^*:=\frac{Qp}{Q-ps}$. Next, we will discuss several definitions which would be crucial for the proof of Theorem 1.1 below. We begin by defining the notion of a weak solution for the nonlocal equation (4.1).

### Definition 4.1

We say that $u \in W_{0}^{s,p}(\mathbb {G})$ is a weak solution of (4.1) if and only if for all $\phi \in W_{0}^{s,p}( \mathbb {G})$, we have

4.2 $$\begin{aligned}&\int _{\mathbb {G}}\int _{\mathbb {G}}\frac{|u(x)-u(y)|^{p-2}(u(x)-u(y))(\phi (x)-\phi (y)}{|y^{-1}x|^{Q+ps}}dxdy\nonumber \\  &\qquad + \int _{ \mathbb {G}}\left( |u|^{p-2}u\phi -|u|^{q-2} u \phi \right) dx=0. \end{aligned}$$

The energy functional $I: W^{s,p}_0(\mathbb G) \rightarrow \mathbb {R}$ associated to the problem (4.1) is defined by

$$\begin{aligned} I(u):=\frac{1}{p} \iint _{\mathbb G\times \mathbb G} \frac{|u(x)-u(y)|^p}{|y^{-1} x|^{Q+ps}} dx dy +\frac{1}{p} \int _{\mathbb G} |u(x)|^{p} dx -\frac{1}{q} \int _{ \mathbb G}|u(x)|^{q} dx \end{aligned}$$

such that

$$\begin{aligned} \langle I'(u),\phi \rangle&= \int _{\mathbb {G}}\int _{\mathbb {G}}\frac{|u(x)-u(y)|^{p-2}(u(x)-u(y))(\phi (x)-\phi (y)}{|y^{-1}x|^{Q+ps}}dxdy \\&\quad \quad \quad + \int _{ \mathbb {G}}\left( |u|^{p-2}u\phi -|u|^{q-2} u \phi \right) dx, \end{aligned}$$

for every $\phi \in W^{s,p}_0(\mathbb G).$ In particular, by setting $\mathcal {L}(u):=\langle I'(u), u \rangle ,$ we get

$$\begin{aligned} \mathcal {L}(u):=\int _{\mathbb {G}}\int _{\mathbb {G}}\frac{|u(x)-u(y)|^{p}}{|y^{-1}x|^{Q+ps}}dxdy + \int _{ \mathbb {G}}\left( |u|^{p}-|u|^{q} \right) dx. \end{aligned}$$

Denote the Nehari manifold by

4.3 $$\begin{aligned} \mathcal {N}:=\left\{ u \in W_{0}^{s,p}(\mathbb G) \setminus \{0\}: \mathcal {L}(u)=\langle I'(u),u\rangle =0\right\} \end{aligned}$$

and set

4.4 $$\begin{aligned} d:=\inf \{I(u): u \in \mathcal {N}\} . \end{aligned}$$

Since it is clear that the weak solutions of (4.1) are the critical points of the energy functional *I*, we define the following set:

### Definition 4.2

Let $\Gamma$ be the set of weak solutions of (4.1), namely, we define

$$\begin{aligned} \Gamma :=\left\{ \phi \in W_{0}^{s,p}( \mathbb G): I'(\phi )=0 \text{ and } \phi \ne 0\right\} . \end{aligned}$$

Let $\mathcal {G}$ be the set of least energy solutions of (4.1), that is,

$$\begin{aligned} \mathcal {G}:=\{u \in \Gamma : I(u) \le I(v) \text{ for } \text{ any } v \in \Gamma \}. \end{aligned}$$

The following supporting lemmas will be useful in what follows.

### Lemma 4.3

For any $u \in W_{0}^{s,p}( \mathbb G) \setminus \{0\}$, there is a unique $\theta _{u}>0$ such that $\theta _{u} u \in$ $\mathcal {N}$. Moreover, if $\mathcal {L}(u)<0$, then $0<\theta _{u}<1$.

### Proof

For any arbitrary $u \in W_{0}^{s,p}( \mathbb G) \setminus \{0\}$ and

$$\begin{aligned} \theta _{u}=\Vert u\Vert _{W_{0}^{s,p}( \mathbb G)}^{\frac{p}{q-p}}\Vert u\Vert _{L^{q}(\mathbb G)}^{-\frac{q}{q-p}}. \end{aligned}$$

A simple calculation yields that $\theta _{u} u \in \mathcal {N}$. It is easy to see that this $\theta _{u}$ is unique. From above and the expression of $\mathcal {L}(u)=\Vert u\Vert _{W_{0}^{s,p}( \mathbb G)}^{p}-\Vert u\Vert _{L^{q}(\mathbb G)}^{q}$, we know that if $\mathcal {L}(u)<0$, that is, $\Vert u\Vert _{W_{0}^{s,p}( \mathbb G)}^{p}<\Vert u\Vert _{L^{q}(\mathbb G)}^{q}$, then $0<\theta _{u}<1$. $\square$

### Lemma 4.4

There is $\rho >0$ such that for all $u \in \mathcal {N}$ we have $\Vert u\Vert _{W_{0}^{s,p}( \mathbb G)} \ge \rho$.

### Proof

Since $u \in \mathcal {N},$ we have $\Vert u\Vert _{W_{0}^{s,p}( \mathbb G)}^{p}=\Vert u\Vert _{L^{q}(\mathbb G)}^{q}.$ Therefore, from the subelliptic fractional Gagliardo-Nirenberg inequality (1.19) and subelliptic fractional Sobolev inequality (2.14) we obtain

$$\begin{aligned} \Vert u\Vert _{W_{0}^{s,p}( \mathbb G)}^{p}&=\Vert u\Vert _{L^{q}(\mathbb G)}^{q} \le C\left( \int _{\mathbb {G}}\int _{\mathbb {G}}\frac{|u(x)-u(y)|^{p}}{\left| y^{-1} x\right| ^{Q+ps}} dxdy\right) ^{\frac{Q(q-p)}{sp^2}}\left( \int _{ \mathbb {G}}|u|^{p} dx\right) ^{\frac{spq-Q(q-p)}{sp^2}} \\  &\le \Vert u\Vert _{W^{s,p}_0(\mathbb G)}^{\frac{Q(q-p)}{sp}} \Vert u\Vert _{L^p(\mathbb G)}^{\frac{spq-Q(q-p)}{sp}} \le C_{Q, s, p}\Vert u\Vert _{W^{s,p}_0(\mathbb G)}^{\frac{Q(q-p)}{sp}} \Vert u\Vert _{W^{s,p}_0(\mathbb G)}^{\frac{spq-Q(q-p)}{sp}}\\  &= C_{Q, s, p} \Vert u\Vert _{W^{s,p}_0(\mathbb G)}^{q}, \end{aligned}$$

which implies that $\Vert u\Vert _{W^{s,p}_0(\mathbb G)}^{p-q} \ge C_{Q, s, p}^{-1}$. Setting $\rho =C_{Q, s, p}^{-\frac{1}{p-q}}>0$, we get that $\Vert u\Vert _{W^{s,p}_0(\mathbb G)} \ge \rho$ for all $u \in \mathcal {N}$. $\square$

### Lemma 4.5

Suppose that $v \in \mathcal {N}$ and $I(v)=d:=\inf \{I(u): u \in \mathcal {N}\}.$ Then *v* is a least energy solution of (4.1).

### Proof

Let $I(v)=d$ for $v \in \mathcal {N}.$ This implies that *v* is a minimizer for *d*. Therefore, from the Lagrange multiplier rule, we infer that there is $\theta \in \mathbb {R}$ such that for any $\psi \in W_{0}^{s,p}( \mathbb G)$,

4.5 $$\begin{aligned} \Big \langle I'(v), \psi \Big \rangle _{\mathbb G}=\theta \left\langle \mathcal {L}^{'}(v), \psi \right\rangle _{\mathbb G}. \end{aligned}$$

Here $\langle \cdot , \cdot \rangle _{\mathbb G}$ is a dual product between $W^{s,p}_0(\mathbb G)$ and its dual space. Note that as $q>p$ we conclude, noting that $v \in \mathcal {N}$ and so $\mathcal {L}(v)=\Vert u\Vert _{W_{0}^{s,p}( \mathbb G)}^{p}-\Vert u\Vert _{L^{q}(\mathbb G)}^{q}=0,$ that

$$\begin{aligned} \left\langle \mathcal {L}^{'}(v), v\right\rangle _{\mathbb G}=p\Vert v\Vert _{W^{s,p}_0(\mathbb G)}^{p}-q \int _{\mathbb G}|v|^{q} dx=(p-q) \int _{\mathbb G}|v|^{q} dx<0. \end{aligned}$$

In addition, $\left\langle I'(v), v\right\rangle _{\mathbb G}=\mathcal {L}(v)=0$ by the choice of *v*. Consequently, (4.5) forces that $\theta =0$. Hence $I'(v)=0$. From Definition 4.2, one sees easily that *v* is a least energy solution of (4.1). $\square$

Having these supporting lemmas in hand, we are ready to prove Theorem 1.1.

### Proof of Theorem 1.1

Let us choose $\left( v_{n}\right) \subset \mathcal {N}$ as a minimizing sequence. Then, from the Ekeland variational principle, we deduce that there is a sequence $\left( u_{n}\right) \subset \mathcal {N}$ such that

$$\begin{aligned} I(u_{n}) \rightarrow d \quad \text{ and } \mathcal {L}(u_{n}) \rightarrow 0 . \end{aligned}$$

The subellitic fractional Sobolev inequality (2.14) and Lemma 4.4 imply that there exist two positive constants $C_{1}$ and $C_{2}$ such that

$$\begin{aligned} C_{1} \le \left\| u_{n}\right\| _{W^{s, p}_0(\mathbb G)} \le C_{2} . \end{aligned}$$

Combining this with the fact that $\left\| u_{n}\right\| _{W^{s, p}_0(\mathbb G)}^{p}=\int _{\mathbb G}\left| u_{n}\right| ^{q} dx$ as $(u_n)_n \subset \mathcal {N}$, we have that there exists a positive constant $C_{3}$ such that

4.6 $$\begin{aligned} \limsup _{n \rightarrow \infty } \int _{\mathbb G}\left| u_{n}(x)\right| ^{q} dx \ge C_{3}>0 . \end{aligned}$$

Using the subelliptic Lions vanishing lemma (see Lemma 3.1), we have that $u_{n} \rightarrow 0$ in $L^{q}\left( \mathbb G\right)$ for any $p<q<p_s^*$ provided that

$$\begin{aligned} \liminf _{n \rightarrow \infty } \sup _{y \in \mathbb G} \int _{B(y, 1)}\left| u_{n}(x)\right| ^{q} dx=0. \end{aligned}$$

Therefore, (4.6) implies that there exists $C_{4}>0$ such that

4.7 $$\begin{aligned} \liminf _{n \rightarrow \infty } \sup _{y \in \mathbb G} \int _{B(y, 1)}\left| u_{n}(x)\right| ^{q} dx \ge C_{4}>0 . \end{aligned}$$

Therefore, we may assume that there is a $\tilde{y} \in \mathbb G$ such that

4.8 $$\begin{aligned} \liminf _{n \rightarrow \infty } \int _{B\left( \tilde{y}, 1\right) }\left| u_{n}(x)\right| ^{p} dx \ge \frac{C_{4}}{2}>0 . \end{aligned}$$

Using the bi-invariance property of the Haar measure *dx* on $\mathbb G,$ a direct computation yields that

$$\begin{aligned} I(u_{n}(y \circ \tilde{y}))=I(u_{n}(y)) \end{aligned}$$

and

$$\begin{aligned} \mathcal {L}(u_{n}(y \circ \tilde{y}))=\mathcal {L}(u_{n}(y)) \end{aligned}$$

for all $\tilde{y} \in \mathbb G.$ Let us define a new sequence $w_{n}(y):=u_{n}(y \circ \tilde{y})$. It is clear that $I(w_{n})=I(u_{n})$ and $\mathcal {L}(w_{n})=\mathcal {L}(u_{n})$. Moreover, it provides that $(w_{n})$ is bounded in $W_{0}^{s,p}( \mathbb G)$ satisfying

4.9 $$\begin{aligned} \liminf _{n \rightarrow \infty } \int _{B(0, 1)}\left| w_{n}(x)\right| ^{q} dx \ge \frac{C_{4}}{2}>0 . \end{aligned}$$

Passing to a subsequence we may assume $w_{n} \rightarrow \phi$ weakly in $W_{0}^{s,p}( \mathbb G)$. Theorem 2.4 implies that $w_{n} \rightarrow \phi$ strongly in $L_{\text {loc}}^{q}( \mathbb G)$. Combining this with (4.9), we know that $\phi \ne 0$.

Next we will show that $(w_{n})$ converges to $\phi$ strongly in $W_{0}^{s,p}( \mathbb G)$. For this purpose, we first prove that $\mathcal {L}(\phi )=0.$ Contrary to this, let us consider the first case, if possible, $\mathcal {L}(\phi )<0$. Then, Lemma 4.3 immediately implies that there exits a $0<\theta _{\phi }<1$ such that $\theta _{\phi } \phi \in \mathcal {N}$. Therefore using the Fatou’s lemma and the fact that $\mathcal {L}(w_{n})=0$, we obtain

4.10 $$\begin{aligned} d+o(1)&=I(w_{n})=\left( 1-\frac{1}{q}\right) \int _{\mathbb G}\left| w_{n}\right| ^{q} dx -\left( 1-\frac{1}{p}\right) \int _{\mathbb G}\left| w_{n}\right| ^{p} dx \nonumber \\&\ge \left( 1-\frac{1}{q}\right) \int _{\mathbb G}\left| w_{n}\right| ^{q} dx \nonumber \\&\ge \left( 1-\frac{1}{q}\right) \int _{\mathbb G}\left| \phi \right| ^{q} dx +o(1) =\left( 1-\frac{1}{q}\right) \theta _{\phi }^{-q} \int _{\mathbb G}\left| \theta _{\phi } \phi \right| ^{q} dx+o(1)\nonumber \\&\ge \left( 1-\frac{1}{q}\right) \theta _{\phi }^{-q} \int _{\mathbb G}\left| \theta _{\phi } \phi \right| ^{q} dx -\left( 1-\frac{1}{p}\right) \theta _{\phi }^{-q} \int _{\mathbb G}\left| \theta _{\phi }\phi \right| ^{p} dx +o(1) \nonumber \\&=\theta _{\phi }^{-q} I(\theta _{\phi } \phi )+o(1) . \end{aligned}$$

We now deduce from $0<\theta _{\phi }<1$ that $I(\theta _{\phi } \phi )<d$, which is a contradiction as $\theta _{\phi } \phi \in \mathcal {N}$.

Now, we consider the remaining case, $\mathcal {L}(\phi )>0$. Then, from Brezis-Lieb lemma [15, Theorem 1.3.5] with $\psi _{n}=w_{n}-\phi$ we have that

$$\begin{aligned} 0=\mathcal {L}(w_{n})=\mathcal {L}(\phi )+\mathcal {L}(\psi _{n})+o(1). \end{aligned}$$

As a consequence, along with the fact that $\mathcal {L}(\phi )>0$, one deduces that

4.11 $$\begin{aligned} \limsup _{n \rightarrow \infty } \mathcal {L}\left( \psi _{n}\right) <0 . \end{aligned}$$

By Lemma 4.3, there is positive constant $\theta _{n}:=\theta _{\psi _{n}}$ such that $\theta _{n} \psi _{n} \in \mathcal {N}$. Moreover, we claim that $\lim \sup _{n \rightarrow \infty } \theta _{n} \in (0,1)$. Indeed, suppose that $\lim \sup _{n \rightarrow \infty } \theta _{n}=1$. Then, we can extract a subsequence $\left( \theta _{n_{j}}\right)$ such that $\lim _{j \rightarrow \infty } \theta _{n_{j}}=1$. Thus from the property $\theta _{n_{j}} \psi _{n_{j}} \in \mathcal {N}$, we obtain that $\mathcal {L}\left( \psi _{n_{j}}\right) =\mathcal {L}\left( \theta _{n_{j}} \psi _{n_{j}}\right) +o(1)=o(1)$. This is not possible in view of (4.11). Therefore, we obtain the required conclusion that $\underset{n \rightarrow \infty }{\limsup }\, \theta _{n} \in (0,1)$. Since $\mathcal {L}(w_n)=0,$ similar to (4.10), using the Fatou’s lemma we get

$$\begin{aligned} \begin{aligned} d+o(1)&=I\left( w_{n}\right) = \left( 1-\frac{1}{q}\right) \int _{\mathbb G}\left| w_{n}\right| ^{q} dx -\left( 1-\frac{1}{p}\right) \int _{\mathbb G}\left| w_{n}\right| ^{p} dx \\&\ge \left( 1-\frac{1}{q}\right) \int _{\mathbb G}\left| w_{n}\right| ^{q} dx \ge \left( 1-\frac{1}{q}\right) \int _{\mathbb G}\left| \psi _{n}\right| ^{q} dx+o(1) \\&\ge \left( 1-\frac{1}{q}\right) \theta _{n}^{-q} \int _{\mathbb G}\left| \theta _{n} \psi _{n}\right| ^{q} dx-\left( 1-\frac{1}{p}\right) \theta _{n}^{-q} \int _{\mathbb G}\left| \theta _{n} \psi _{n}\right| ^{p} dx+o(1) \\  &=\theta _{n}^{-q} I\left( \theta _{n} \psi _{n}\right) +o(1). \end{aligned} \end{aligned}$$

This along with the fact that $\underset{n \rightarrow \infty }{\lim \sup }\, \theta _{n} \in (0,1)$ implies that $d>I\left( \theta _{n} \psi _{n}\right)$, which is a contradiction as $\theta _{n} \psi _{n} \in \mathcal {N}$.

By joining both cases together, we conclude that $\mathcal {L}(\phi )=0$.

We are now in a position to prove our claim that $\psi _{n}=w_n-\phi \rightarrow 0$ in $W_{0}^{s,p}( \mathbb G)$. Indeed, if possible, suppose that it is not true, that means, $\left\| \psi _{n}\right\| _{W^{s,p}(\mathbb G)} \nrightarrow 0$ as $n \rightarrow \infty$. Then, as an application of the continuous fractional Sobolev embedding (2.14), the following two situations are possible:

(i) When $\int _{\mathbb G}\left| \psi _{n}\right| ^{q} dx \nrightarrow 0$ as $n \rightarrow \infty ,$ we have from

$$\begin{aligned} 0=\mathcal {L}\left( w_{n}\right) =\mathcal {L}(\phi )+\mathcal {L}\left( \psi _{n}\right) +o(1)=\mathcal {L}\left( \psi _{n}\right) +o(1) \end{aligned}$$

and therefore, $\psi _n \in \mathcal {N}.$ The Brezis-Lieb lemma [15, Theorem 1.3.5] implies that

$$\begin{aligned} d+o(1)=I\left( w_{n}\right) =I(\phi )+I\left( \psi _{n}\right) +o(1) \ge d+d+o(1), \end{aligned}$$

which is not true as $d>0$.

(ii) When $\int _{\mathbb G}\left| \psi _{n}\right| ^{q} dx \rightarrow 0$ as $n \rightarrow \infty ,$ we have that

$$\begin{aligned} d+o(1)=I\left( w_{n}\right) =I(\phi )+\frac{1}{p}\left\| \psi _{n}\right\| _{W^{s,p}_0(\mathbb G)}^{p}+o(1) \ge d+\frac{1}{p}\left\| \psi _{n}\right\| _{W^{s,p}_0(\mathbb G)}^{p}+o(1)>d \end{aligned}$$

which is impossible. Thus, our assumption that $\left\| \psi _{n}\right\| _{W^{s,p}(\mathbb G)} \nrightarrow 0$ is not correct. Therefore, we deduce that $(w_{n})$ converges strongly to $\phi$ in $W_{0}^{s,p}( \mathbb G)$ and $\phi$ is a minimizer for *d*. Finally, Lemma 4.5 implies that $\phi$ is a least energy solution of (4.1). This completes the proof.

## Best constants in the subelliptic fractional Gagliardo-Nirenberg inequalities

Throughout this section, we continue to assume the same range of *q*,  that is, $p<q<p_s^*$, as in the previous section. The aim of this section is to give a sharp estimate of $C_{GN, \mathbb G}$ in (1.19). Firstly, we give some properties of the least energy solutions of (4.1) which will be used in what follows.

### Lemma 5.1

Let $\phi$ be a least energy solution of (4.1). Then

5.1 $$\begin{aligned} \iint _{\mathbb {G} \times \mathbb {G}}\frac{|\phi (x)-\phi (y)|^{p}}{|y^{-1}x|^{Q+ps}}dxdy=\frac{Q(q-p)}{pqs-Q(q-p)} \int _{\mathbb G}|\phi |^{p} dx \end{aligned}$$

and

5.2 $$\begin{aligned} \int _{\mathbb G}|\phi |^{q} dx=\frac{pqs}{pqs-Q(q-p)} \int _{\mathbb G}|\phi |^{p} dx. \end{aligned}$$

### Proof

Since $\phi$ is a least energy solution of (4.1), we obtain from (4.2) that

5.3 $$\begin{aligned} \iint _{\mathbb {G} \times \mathbb {G}}\frac{|\phi (x)-\phi (y)|^{p}}{|y^{-1}x|^{Q+ps}}dxdy+\int _{\mathbb G} |\phi (x)|^{p} dx=\int _{\mathbb G}|\phi (x)|^{q} dx. \end{aligned}$$

Define a function $\tilde{\phi }_{\lambda }$ for $\lambda >0$ by $\tilde{\phi }_{\lambda }(x):=\lambda ^{\frac{Q}{p}} \phi \left( D_{\lambda }(x)\right)$. Then, we have

$$\begin{aligned} I(\tilde{\phi }_{\lambda })&= \frac{\lambda ^Q}{p} \iint _{\mathbb G\times \mathbb G} \frac{|\tilde{\phi }_{\lambda }(x)-\tilde{\phi }_{\lambda }(y)|^p}{|y^{-1} x|^{Q+ps}} dx dy +\frac{\lambda ^Q}{p} \int _{\mathbb G} |\tilde{\phi }_{\lambda }(x)|^{p} dx -\frac{\lambda ^{\frac{Qq}{p}}}{q} \int _{ \mathbb G}|\tilde{\phi }_{\lambda }(x)|^{q} dx \\&= \frac{\lambda ^{sp}}{p} \iint _{\mathbb G\times \mathbb G} \frac{|\phi (x)-\phi (y)|^p}{|y^{-1} x|^{Q+ps}} dx dy +\frac{1}{p} \int _{\mathbb G} |\phi (x)|^{p} dx -\frac{\lambda ^{\frac{Qq}{p}-Q}}{q} \int _{ \mathbb G}|\phi (x)|^{q} dx. \end{aligned}$$

Then, it follows that

5.4 $$\begin{aligned} 0=\left. \frac{\partial }{\partial \lambda } I\left( \tilde{\phi }_{\lambda }\right) \right| _{\lambda =1}= s\iint _{\mathbb G\times \mathbb G} \frac{|\phi (x)-\phi (y)|^p}{|y^{-1} x|^{Q+ps}} dx dy -\frac{Q(q-p)}{pq} \int _{ \mathbb G}|\phi (x)|^{q} dx. \end{aligned}$$

Using (5.4) in (5.3) we get that

$$\begin{aligned} \int _{\mathbb G} |\phi (x)|^p dx = \Bigg (1 -\frac{Q(q-p)}{spq} \Bigg ) \int _{ \mathbb G}|\phi (x)|^{q} dx, \end{aligned}$$

which implies that

5.5 $$\begin{aligned} \int _{\mathbb G}|\phi |^{q} dx=\frac{pqs}{pqs-Q(q-p)} \int _{\mathbb G}|\phi |^{p} dx \end{aligned}$$

establishing (5.2). Using this in (5.3), we get

$$\begin{aligned}\int _{\mathbb {G}}\int _{\mathbb {G}}\frac{|\phi (x)-\phi (y)|^{p}}{|y^{-1}x|^{Q+ps}}dxdy&=\Bigg (\frac{pqs}{pqs-Q(q-p)}-1 \Bigg ) \int _{\mathbb G}|\phi |^{p} dx\\  &= \frac{Q(p-q)}{pqs-Q(q-p)} \int _{\mathbb G}|\phi |^{p} dx,\end{aligned}$$

proving (5.1). $$\square $$

### Lemma 5.2

Let

$$\begin{aligned} T_{\rho ,p,q,s}:=\inf \left\{ \Vert u\Vert ^p_{W^{s,p}_0(\mathbb G)}: u \in W_{0}^{s,p}\left( \mathbb G\right) ~ \text { and }~ \int _{\mathbb G}|u|^{q} dx=\rho \right\} . \end{aligned}$$

If $\phi$ is a minimizer obtained in Theorem 1.1, then $\phi$ is a minimizer of $T_{\rho _{0}, p,q, s}$ with $\rho _{0}=\int _{\mathbb G}|\phi |^{q} dx$.

### Proof

It is clear from the definition of $T_{\rho ,p,q,s}$ that $\Vert \phi \Vert ^{p}_{W^{s,p}_0(\mathbb G)} \ge T_{\rho _{0},p,q,s}$. Now, for any $u \in W_{0}^{s,p}\left( \mathbb G\right)$ satisfying $\int _{\mathbb G}|u|^{q} dx=$ $\int _{\mathbb G}|\phi |^{q} dx$, by Lemma 4.3 there is a unique

$$\begin{aligned} \lambda _{0}=\Vert u\Vert _{W^{s,p}_0(\mathbb G)}^{\frac{p}{q-p}}\left( \int _{\mathbb G}|u|^{q} dx\right) ^{-\frac{1}{q-p}} \end{aligned}$$

such that $\lambda _{0} u \in \mathcal {N},$ which implies that $\mathcal {L}\left( \lambda _{0} u\right) =0$. Since $\lambda _{0} u \ne 0$ and $\phi$ achieves the minimum *d*, a simple calculation asserts that

5.6 $$\begin{aligned} \left( \frac{1}{p}-\frac{1}{q}\right) \Vert \phi \Vert ^{p}_{W^{s,p}_0(\mathbb G)}&=I(\phi ) \le I\left( \lambda _{0} u\right) =\left( \frac{1}{p}-\frac{1}{q}\right) \lambda _{0}^{p}\Vert u\Vert ^{p}_{W^{s,p}_0(\mathbb G)} \nonumber \\&=\left( \frac{1}{p}-\frac{1}{q}\right) \Vert u\Vert _{W^{s,p}_0(\mathbb G)}^{\frac{p^2}{q-p}}\left( \int _{\mathbb G}|u|^{q} dx\right) ^{-\frac{p}{q-p}}\Vert u\Vert _{W^{s,p}_0(\mathbb G)}^{p}\nonumber \\&=\left( \frac{1}{p}-\frac{1}{q}\right) \Vert u\Vert _{W^{s,p}_0(\mathbb G)}^{\frac{pq}{q-p}} \left( \int _{\mathbb G}|u|^{q} dx\right) ^{-\frac{p}{q-p}}. \end{aligned}$$

Since $\int _{\mathbb G}|u|^{q} dx=\int _{\mathbb G}|\phi |^{q} dx$ and $\int _{\mathbb G}|\phi |^{q} dx=\Vert \phi \Vert ^{p}_{W^{s,p}_0(\mathbb G)},$ we get that

5.7 $$\begin{aligned} \left( \frac{1}{p}-\frac{1}{q}\right) \Vert \phi \Vert ^{p}_{W^{s,p}_0(\mathbb G)}&\le \left( \frac{1}{p}-\frac{1}{q}\right) \Vert u\Vert _{W^{s,p}_0(\mathbb G)}^{\frac{pq}{q-p}} \Vert \phi \Vert _{W^{s,p}_0(\mathbb G)}^{-\frac{p^2}{q-p}} \end{aligned}$$

implying that

$$\begin{aligned} \Vert \phi \Vert ^{p}_{W^{s,p}_0(\mathbb G)} \le \Vert u\Vert ^{p}_{W^{s,p}_0(\mathbb G)}. \end{aligned}$$

Since *u* is chosen arbitrarily, we get that $T_{\rho _{0},p,q,s} \ge \Vert \phi \Vert ^{p}_{W^{s,p}_0(\mathbb G)}$. Therefore, we have $T_{\rho _{0},p,q,s}=\Vert \phi \Vert ^{p}_{W^{s,p}_0(\mathbb G)}$ and this show that $\phi$ is a minimizer of $T_{\rho _{0},p,q,s}$. $\square$

Now we are ready to obtain an expression for $C_{GN, \mathbb G}$. In other words, we will now present a proof of Theorem 1.3.

### Proof of Theorem 1.3

We define the functional, for all $u \in W^{s,p}_0(\mathbb G) \backslash \{0\},$

$$\begin{aligned} J(u):=\frac{\left( \int _{\mathbb {G}}\int _{\mathbb {G}}\frac{|u(x)-u(y)|^{p}}{\left| y^{-1} x\right| ^{Q+ps}} dxdy\right) ^{\frac{Q(q-p)}{sp^2}}\left( \int _{\mathbb {G}}|u(x)|^{p}dx\right) ^{\frac{spq-Q(q-p)}{sp^2}}}{\int _{\mathbb {G}}|u(x)|^{q}dx}. \end{aligned}$$

Then the sharp estimate of $C_{GN, \mathbb G}$ can be estimated by studying the following minimization problem

$$\begin{aligned} C_{GN, \mathbb G}^{-1}=\inf \left\{ J(u): u \in W_{0}^{s,p}\left( \mathbb G\right) \backslash \{0\}\right\} . \end{aligned}$$

Let $\phi \in W^{s,p}_0(\mathbb G)$ be a least energy solution of (1.3). Then, by the definition of *J*, we obtain

$$\begin{aligned} C_{GN, \mathbb G}^{-1} \le J(\phi ). \end{aligned}$$

From Lemma 5.1 we obtain that

$$\begin{aligned} C_{GN, \mathbb G}^{-1} \le J(\phi )=&\frac{\left( \int _{\mathbb {G}}\int _{\mathbb {G}}\frac{|\phi (x)-\phi (y)|^{p}}{\left| y^{-1} x\right| ^{Q+ps}} dxdy\right) ^{\frac{Q(q-p)}{sp^2}}\left( \int _{\mathbb {G}}|\phi (x)|^{p}dx\right) ^{\frac{spq-Q(q-p)}{sp^2}}}{\int _{\mathbb {G}}|\phi (x)|^{q}dx}\\ =&\frac{\left( \frac{Q(q-p)}{pqs-Q(q-p)} \int _{\mathbb G}|\phi |^{p} dx\right) ^{\frac{Q(q-p)}{sp^2}}\left( \int _{\mathbb {G}}|\phi (x)|^{p}dx\right) ^{\frac{spq-Q(q-p)}{sp^2}}}{\frac{pqs}{pqs-Q(q-p)} \int _{\mathbb G}|\phi |^{p} dx} \\ =&\frac{pqs-Q(q-p)}{pqs} \left( \frac{Q(q-p)}{pqs-Q(q-p)} \right) ^{\frac{Q(q-p)}{sp^2}}\left( \int _{\mathbb G}|\phi |^{p} dx\right) ^{\frac{q-p}{p}}. \end{aligned}$$

Now, we will estimate $C_{GN, \mathbb G}^{-1}$ from below. For any $u \in W_{0}^{s,p}\left( \mathbb G\right) \backslash \{0\}$, we define

$$\begin{aligned} w(x):=\lambda u\left( D_{\mu }(x)\right) , \end{aligned}$$

where $\lambda$ and $\mu$ are two positive parameters, which will be determined later. It is easy to see, by direct calculations, that

5.8 $$\begin{aligned} \int _{\mathbb {G}}\int _{\mathbb {G}}\frac{|w(x)-w(y)|^{p}}{|y^{-1}x|^{Q+ps}}dxdy=\lambda ^{p} \mu ^{ps-Q} \int _{\mathbb {G}}\int _{\mathbb {G}}\frac{|u(x)-u(y)|^{p}}{|y^{-1}x|^{Q+ps}}dxdy, \end{aligned}$$

5.9 $$\begin{aligned} \int _{\mathbb G}|w(x)|^{p} dx=\lambda ^{p} \mu ^{-Q} \int _{\mathbb G}|u(x)|^{p} dx \end{aligned}$$

and

5.10 $$\begin{aligned} \int _{\mathbb G}|w(x)|^{q} dx=\lambda ^{q} \mu ^{-Q} \int _{\mathbb G}|u(x)|^{q} dx. \end{aligned}$$

We choose $\lambda$ and $\mu$ such that

$$\begin{aligned} \lambda ^{q} \mu ^{-Q} \int _{\mathbb G}|u(x)|^{q} dx:=\int _{\mathbb G}|\phi (x)|^{q} dx=\frac{pq s}{p qs-Q(q-p)} \int _{\mathbb G}|\phi (x)|^{p} dx \end{aligned}$$

and

$$\begin{aligned} \lambda ^{p} \mu ^{-Q} \int _{\mathbb G}|u(x)|^{p} dx:=\int _{\mathbb G}|\phi (x)|^{p} dx. \end{aligned}$$

From the above two equalities, it is straightforward to see that

$$\begin{aligned} \lambda ^{p}=\left( \frac{pqs}{ pqs-Q(q-p)}\right) ^{\frac{p}{q-p}}\left( \int _{\mathbb G}|u|^{p} dx\right) ^{\frac{p}{q-p}}\left( \int _{\mathbb G}|u|^{q} dx\right) ^{-\frac{p}{q-p}} \end{aligned}$$

and

$$\begin{aligned} \mu ^{ps-Q}=&\left( \frac{ pqs}{pqs-Q(q-p)}\right) ^{\frac{p}{q-p} \cdot \frac{ps-Q}{Q}}\left( \int _{\mathbb G}|u|^{p} dx\right) ^{\frac{q}{q-p} \cdot \frac{ps-Q}{Q}} \\&\quad \quad \quad \times \left( \int _{\mathbb G}|u|^{q} dx\right) ^{-\frac{p}{q-p} \cdot \frac{ps-Q}{Q}}\left( \int _{\mathbb G}|\phi |^{p} dx\right) ^{-\frac{ps-Q}{Q}}. \end{aligned}$$

From the choice of the parameters $\lambda$ and $\mu$ as above and from (5.9) and (5.10), we have that

$$\begin{aligned} \int _{\mathbb G}|w|^{p} dx=\int _{\mathbb G}|\phi |^{p} dx \end{aligned}$$

and

$$\begin{aligned} \int _{\mathbb G}|w|^{q} dx=\int _{\mathbb G}|\phi |^{q} dx. \end{aligned}$$

Since $\phi$ is a minimizer of $T_{\rho _{0},p,q,s}$ with $\rho _{0}=\int _{\mathbb G}|\phi |^{q} dx$, by Lemma 5.2 we get that

$$\begin{aligned} \int _{\mathbb {G}}\int _{\mathbb {G}}\frac{|w(x)-w(y)|^{p}}{|y^{-1}x|^{Q+ps}}dxdy \ge \int _{\mathbb {G}}\int _{\mathbb {G}}\frac{|\phi (x)-\phi (y)|^{p}}{|y^{-1}x|^{Q+ps}}dxdy. \end{aligned}$$

Therefore, using (5.8) and by Lemma 5.1 we get

$$\begin{aligned} \lambda ^{p} \mu ^{ps-Q}&\int _{\mathbb {G}}\int _{\mathbb {G}}\frac{|u(x)-u(y)|^{p}}{|y^{-1}x|^{Q+ps}}dxdy=\int _{\mathbb {G}}\int _{\mathbb {G}}\frac{|w(x)-w(y)|^{p}}{|y^{-1}x|^{Q+ps}}dxdy\\&\ge \int _{\mathbb {G}}\int _{\mathbb {G}}\frac{|\phi (x)-\phi (y)|^{p}}{|y^{-1}x|^{Q+ps}}dxdy=\frac{Q(q-p)}{pqs-Q(q-p)} \int _{\mathbb G}|\phi |^{p} dx. \end{aligned}$$

Therefore, by substituting the value of $\lambda ^p$ and $\mu ^{ps-Q}$ we have

$$\begin{aligned}&\left( \frac{pqs}{ pqs-Q(q-p)}\right) ^{\frac{p}{q-p}\left( 1+\frac{ps-Q}{Q}\right) }\left( \int _{\mathbb G}|u|^{p} dx\right) ^{\frac{p}{q-p}+\frac{q}{q-p} \cdot \frac{ps-Q}{Q}}\left( \int _{\mathbb G}|u|^{q} dx\right) ^{-\frac{p}{q-p}\left( 1+\frac{ps-Q}{Q}\right) } \\&\quad \times \left( \int _{\mathbb G}|\phi |^{p} dx\right) ^{-\frac{ps-Q}{Q}} \int _{\mathbb {G}}\int _{\mathbb {G}}\frac{|u(x)-u(y)|^{p}}{|y^{-1}x|^{Q+ps}}dxdy \ge \frac{Q(q-p)}{pqs-Q(q-p)} \int _{\mathbb G}|\phi |^{p} dx. \end{aligned}$$

It is now deduced by direct calculations that

$$\begin{aligned} J(u) \ge \frac{pqs-Q(q-p)}{ pqs}\left( \frac{Q(q-p)}{pqs-Q(q-p)}\right) ^{\frac{Q(q-p)}{p^2s}}\left( \int _{\mathbb G}|\phi |^{p} dx\right) ^{\frac{q-p}{p}}. \end{aligned}$$

Since *u* is chosen arbitrarily, we get that

$$\begin{aligned} C_{GN, \mathbb G}^{-1} \ge \frac{pqs-Q(q-p)}{ pqs}\left( \frac{Q(q-p)}{pqs-Q(q-p)}\right) ^{\frac{Q(q-p)}{p^2s}}\left( \int _{\mathbb G}|\phi |^{p} dx\right) ^{\frac{q-p}{p}}. \end{aligned}$$

Therefore, combining the lower and upper estimates of $C_{GN, \mathbb G}^{-1}$ we get (1.21) with the value of $C_{p,q, s, Q}$ given by (1.23).

Now, using the property $d=I(\phi )$ and Lemma 5.1 we have

$$\begin{aligned} d=I(\phi )&=\frac{1}{p} \iint _{\mathbb G\times \mathbb G} \frac{|\phi (x)-\phi (y)|^p}{|y^{-1} x|^{Q+ps}} dx dy +\frac{1}{p} \int _{\mathbb G} |\phi (x)|^{p} dx -\frac{1}{q} \int _{ \mathbb G}|\phi (x)|^{q} dx\\  &=\frac{Q(q-p)}{p^2qs-pQ(q-p)} \int _{\mathbb G}|\phi |^{p} dx+\frac{1}{p} \int _{\mathbb G} |\phi (x)|^{p} dx\\  &\quad -\frac{ps}{pqs-Q(q-p)} \int _{\mathbb G}|\phi |^{p} dx\\&=\frac{(q-p)s}{pqs-Q(q-p)} \int _{\mathbb G}|\phi |^{p} dx. \end{aligned}$$

This implies that

5.11 $$\begin{aligned} \int _{\mathbb G}|\phi |^{p} dx=\frac{pqs-Q(q-p)}{(q-p)s} d, \end{aligned}$$

which, combined with the first equality of (1.21), gives

$$\begin{aligned} C^{-1}_{GN, \mathbb G}=\frac{pqs-Q(q-p)}{pqs}\left( \frac{Q(q-p)}{pqs-Q(q-p)}\right) ^{\frac{Q(q-p)}{sp^2}}\left( \frac{pqs-Q(q-p)}{(q-p)s} d\right) ^{\frac{q-p}{p}}, \end{aligned}$$

proving (1.21) with $C_{p,q, s, Q}$ given by (1.22). Hence, the proof of Theorem 1.3 is completed.

## Best constants on the subelliptic fractional Sobolev inequalities

The purpose of this section is to discuss the sharp constant $C_{S, \mathbb G}$ in the subelliptic fractional Sobolev inequalities on the stratified Lie groups. Here $C_{S, \mathbb G}$ is the smallest constant among all positive constants *C* such that

6.1 $$\begin{aligned} \left( \int _{\mathbb {G}}|u(x)|^{q}dx\right) ^{\frac{p}{q}}\le C\left[ \int _{\mathbb {G}}\int _{\mathbb {G}}\frac{|u(x)-u(y)|^{p}}{\left| y^{-1} x\right| ^{Q+ps}} dxdy+\int _{\mathbb {G}}|u(x)|^{p})dx\right] \end{aligned}$$

holds for any $u \in W_{0}^{s,p}\left( \mathbb G\right)$. Then $C_{S, \mathbb G}$ can be given in the following form:

6.2 $$\begin{aligned} C_{S, \mathbb G}^{-1}:=\inf _{u \in W_{0}^{s,p}\left( \mathbb G\right) \backslash \{0\}} \frac{\left[ \int _{\mathbb {G}}\int _{\mathbb {G}}\frac{|u(x)-u(y)|^{p}}{\left| y^{-1} x\right| ^{Q+ps}} dxdy+\int _{\mathbb {G}}|u(x)|^{p})dx\right] }{\left( \int _{\mathbb {G}}|u(x)|^{q}dx\right) ^{\frac{p}{q}}}. \end{aligned}$$

In the following result, we present an expression for $C_{S, \mathbb G}$ in terms of the least energy solution of (1.3).

### Theorem 6.1

Let $p<q<p_s^*:=\frac{Qp}{Q-ps}$ and $s \in (0, 1)$. Let $\phi$ be a least energy solution of (1.3) and let $C_{S, \mathbb {G}}>0$ be the least positive constant such that the inequality (6.1) is true. Then we have

6.3 $$\begin{aligned} C_{S,\mathbb {G}}^{-1}&=\left( \frac{spq}{spq-Q(q-p)}\int _{\mathbb {G}}|\phi (x)|^{p}dx\right) ^{\frac{q-p}{q}}=\left( \frac{pq}{q-p} d \right) ^{\frac{q-p}{q}}, \end{aligned}$$

where $$d=I(\phi ):=\inf _{u\in \mathcal {N}}I(u)$$.

### Proof

Since $\phi$ is a least energy solution of (4.1), $\mathcal {L}(\phi )=0$ and Lemma 5.1 imply that

$$\begin{aligned} \frac{\left[ \int _{\mathbb {G}}\int _{\mathbb {G}}\frac{|\phi (x)-\phi (y)|^{p}}{\left| y^{-1} x\right| ^{Q+ps}} dxdy+\int _{\mathbb {G}}|\phi (x)|^{p})dx\right] }{\left( \int _{\mathbb {G}}|\phi (x)|^{q}dx\right) ^{\frac{p}{q}}}&=\left( \int _{\mathbb G}|\phi |^{q} dx\right) ^{\frac{q-p}{q}}\\  &=\left( \frac{sp q}{spq-Q(q-p)} \int _{\mathbb G}|\phi |^{p} dx\right) ^{\frac{q-p}{q}}, \end{aligned}$$

which further shows that

6.4 $$\begin{aligned} C_{S, \mathbb G}^{-1} \le \left( \frac{sp q}{spq-Q(q-p)} \int _{\mathbb G}|\phi |^{p} dx\right) ^{\frac{q-p}{q}}. \end{aligned}$$

We define $\tilde{u}(x):=\Vert \phi \Vert _{L^{q}(\mathbb G)}\Vert u\Vert _{L^{q}(\mathbb G)}^{-1} u(x)$ for any $u \in W_{0}^{s,p}\left( \mathbb G\right) \backslash \{0\}$ so that

$$\begin{aligned} \int _{\mathbb G}|\tilde{u}(x)|^{q} dx=\int _{\mathbb G}|\phi (x)|^{q} dx. \end{aligned}$$

Therefore we have from Lemma 5.2 that

6.5 $$\begin{aligned}&\int _{\mathbb {G}}\int _{\mathbb {G}}\frac{|\tilde{u}(x)-\tilde{u}(y)|^{p}}{\left| y^{-1} x\right| ^{Q+ps}} dxdy+\int _{\mathbb {G}}|\tilde{u}(x)|^{p})dx \nonumber \\  &\quad \ge \int _{\mathbb {G}}\int _{\mathbb {G}}\frac{|\phi (x)-\phi (y)|^{p}}{\left| y^{-1} x\right| ^{Q+ps}} dxdy+\int _{\mathbb {G}}|\phi (x)|^{p})dx\nonumber \\&\quad =\frac{sp q}{spq-Q(q-p)} \int _{\mathbb G}|\phi |^{p} dx, \end{aligned}$$

where we have used (5.1) in the last equality. Now, a simple calculation gives that

$$\begin{aligned}&\frac{\left[ \int _{\mathbb {G}}\int _{\mathbb {G}}\frac{|u(x)-u(y)|^{p}}{\left| y^{-1} x\right| ^{Q+ps}} dxdy+\int _{\mathbb {G}}|u(x)|^{p})dx\right] }{\left( \int _{\mathbb {G}}|u(x)|^{q}dx\right) ^{\frac{p}{q}}} \\  &\quad \quad \quad \quad = \Bigg ( \int _{\mathbb {G}}\int _{\mathbb {G}}\frac{|\tilde{u}(x)-\tilde{u}(y)|^{p}}{\left| y^{-1} x\right| ^{Q+ps}} dxdy+\int _{\mathbb {G}}|\tilde{u}(x)|^{p})dx\Bigg ) \Vert \phi \Vert _{L^q(\mathbb G)}^{-p}, \end{aligned}$$

and therefore, using (5.2) and (6.5) we obtain that

6.6 $$\begin{aligned}&\frac{\left[ \int _{\mathbb {G}}\int _{\mathbb {G}}\frac{|u(x)-u(y)|^{p}}{\left| y^{-1} x\right| ^{Q+ps}} dxdy+\int _{\mathbb {G}}|u(x)|^{p})dx\right] }{\left( \int _{\mathbb {G}}|u(x)|^{q}dx\right) ^{\frac{p}{q}}} \nonumber \\ \quad \quad \quad \quad&\ge \frac{sp q}{spq-Q(q-p)} \left( \int _{\mathbb G}|\phi |^{p} dx \right) \left( \int _{\mathbb {G}}|\phi (x)|^{q}dx\right) ^{-\frac{p}{q}}\nonumber \\&= \left( \frac{sp q}{spq-Q(q-p)}\int _{\mathbb {G}}|\phi (x)|^{p}dx\right) ^{\frac{q-p}{q}}. \end{aligned}$$

By the arbitrariness of $u \in W^{s,p}_0(\mathbb G)$ in (6.6) we deduce that

6.7 $$\begin{aligned} C_{S, \mathbb G}^{-1} \ge \left( \frac{sp q}{spq-Q(q-p)}\int _{\mathbb {G}}|\phi (x)|^{p}dx\right) ^{\frac{q-p}{q}}. \end{aligned}$$

Finally, the estimates (6.4) and (6.7) imply the first equality in (6.3). The last equality is an immediate consequence of a substitution (5.11) in the first equality of (6.3). This completes the proof. $\square$

##  Subelliptic fractional logarithmic Sobolev inequalities with explicit constants

The aim of this section is to obtain the subelliptic fractional logarithmic Sobolev inequalities with explicit constants on the stratified Lie groups. We adopt the method developed by Merker [70] (see also [17]). In this regard, we first prove the following result, extending a result of Merker from the Euclidean space to general measure spaces.

### Theorem 7.1

Let $\mathbb {X}$ be a measure space. The following statements are equivalent:

(i) The Hölder type inequality
  7.1 $$\begin{aligned} \Vert u\Vert _{L^r(\mathbb {X})} \le \Vert u \Vert _{L^p(\mathbb {X})}^{a} \Vert u\Vert _{L^q(\mathbb {X})}^{1-a} \end{aligned}$$
  is valid for all $u \in L^p(\mathbb {X}) \cap L^q(\mathbb {X})$ for the parameters *p*, *q* and *r* ranging over $0<p\le r\le q \le \infty$ and satisfying $\frac{1}{r}=\frac{a}{p}+\frac{1-a}{q}$ with $a \in [0, 1].$
(ii) The logarithmic Hölder type inequality
  7.2 $$\begin{aligned} \int _{\mathbb {X}} \frac{|u(x)|^p}{\Vert u\Vert _{L^p(\mathbb {X})}^p} \log \left( \frac{|u(x)|^p}{\Vert u\Vert _{L^p(\mathbb {X})}^p} \right) dx\le \frac{q}{q-p} \log \left( \frac{\Vert u\Vert ^p_{L^q(\mathbb {X})}}{\Vert u\Vert _{L^p(\mathbb {X})}^p} \right) , \end{aligned}$$
  is valid for all $u \in L^p(\mathbb {X}) \cap L^q(\mathbb {X})$ for the parameters *p* and *q* satisfying $0<p <q \le \infty .$

### Proof

We first observe that the validity of (7.1) for the parameters *p*, *q* and *r* ranging over $0<p\le r\le q \le \infty$ and satisfying $\frac{1}{r}=\frac{a}{p}+\frac{1-a}{q}$ with $a \in [0, 1]$ is equivalent to the convexity of the function $\phi : \frac{1}{r} \mapsto \log (\Vert u\Vert _r).$ Indeed, this follows by taking logarithms on both sides of (7.1) to have

$$\begin{aligned} \phi \left( \frac{1}{r} \right) \le a\phi \left( \frac{1}{p} \right) +(1-a) \phi \left( \frac{1}{q} \right) , \end{aligned}$$

with $a \in [0, 1]$ satisfying $\frac{1}{r}=\frac{a}{p}+\frac{1-a}{q}.$ Now, the derivative of the function

$$\begin{aligned} \phi (h):=\log (\Vert u\Vert _{\frac{1}{h}})=h \log \Big ( \int _{\mathbb {X}} |u(x)|^{\frac{1}{h}} dx \Big ) \end{aligned}$$

is given by

$$\begin{aligned} \phi ^{'}(h)= \log \Big ( \int _{\mathbb {X}} |u(x)|^{\frac{1}{h}} dx \Big )- \frac{1}{h} \frac{\int _{\mathbb {X}} |u(x)|^{\frac{1}{h}} \log (|u(x)|)dx}{\int _{\mathbb {X}} |u(x)|^{\frac{1}{h}} dx}. \end{aligned}$$

Since the convexity of $\phi$ on $[0, \infty )$ is equivalent to $\phi ^{'}(h) \ge \frac{\phi (h_1)-\phi (h)}{h_1-h}$ for $h>h_1 \ge 0,$ by taking $h=\frac{1}{p}$ and $h_1=\frac{1}{q}$ the convexity of $\phi$ is equivalent to

7.3 $$\begin{aligned} \log \Big ( \int _{\mathbb {X}} |u(x)|^{p} dx \Big )- p \frac{\int _{\mathbb {X}} |u(x)|^{p} \log (|u(x)|)dx}{\int _{\mathbb {X}} |u(x)|^{p} dx} \ge \frac{qp}{p-q} \log \Big (\frac{\Vert u\Vert _{L^q(\mathbb {X})}}{\Vert u\Vert _{L^p(\mathbb {X})}} \Big ) \end{aligned}$$

and therefore, equivalent to

7.4 $$\begin{aligned}&\int _{\mathbb {X}} \frac{|u(x)|^p}{\Vert u\Vert _{L^p(\mathbb {X})}^p} \log \left( \frac{|u(x)|^p}{\Vert u\Vert _{L^p(\mathbb {X})}^p} \right) dx \nonumber \\&= p \frac{\int _{\mathbb {X}} |u(x)|^{p} \log (|u(x)|)dx}{\int _{\mathbb {X}} |u(x)|^{p} dx} -\log \Big ( \int _{\mathbb {X}} |u(x)|^{p} dx \Big ) \le \frac{q}{q-p} \log \left( \frac{\Vert u\Vert ^p_{L^q(\mathbb {X})}}{\Vert u\Vert _{L^p(\mathbb {X})}^p} \right) , \end{aligned}$$

establishing the validity of (7.2) for the range $0<p<q \le \infty$ and its equivalence with (7.1) as this process is reversible at every step. $\square$

The following result follows from Theorem 7.1 by noting that the inequality (7.1) holds for all $1\le p\le r \le q \le \infty$ satisfying $\frac{1}{r}=\frac{a}{q}+\frac{1-a}{q}$ with $a \in [0, 1]$ as a result of the usual Hölder inequality in a measure space. We note here that this result is an improvement of a result established by the third author and collaborators [17] in view of the inclusion of endpoints.

### Theorem 7.2

Let $\mathbb {X}$ be a measure space. Let $u \in L^p(\mathbb {X}) \cap L^q(\mathbb {X}) \backslash \{0\}$ with $1\le p<q\le \infty .$ Then we have

7.5 $$\begin{aligned} \int _{\mathbb {X}} \frac{|u(x)|^p}{\Vert u\Vert _{L^p(\mathbb {X})}^p} \log \left( \frac{|u(x)|^p}{\Vert u\Vert ^p_{L^p(\mathbb {X})}} \right) dx\le \frac{q}{q-p} \log \left( \frac{\Vert u\Vert ^p_{L^q(\mathbb {X})}}{\Vert u\Vert _{L^p(\mathbb {X})}^p} \right) . \end{aligned}$$

The next result is the subelliptic fractional logarithmic Sobolev inequality for inhomogeneous fractional Sobolev spaces on stratified Lie groups.

### Theorem 7.3

Let $s \in (0, 1),$ $p \in (1, \infty ), Q>ps,$ and $p<q<p^*:=\frac{Qp}{Q-ps},$ where *Q* is the homogeneous dimension of a stratified Lie group $\mathbb {G}.$ Then for any $u \ne 0$ we have

7.6 $$\begin{aligned}&\int _{\mathbb G} \frac{|u(x)|^p}{\Vert u\Vert _{L^p(\mathbb G)}^p} \log \left( \frac{|u(x)|^p}{\Vert u\Vert ^p_{L^p(\mathbb G)}} \right) dx \nonumber \\  &\quad \le \frac{Q}{s} \log \left( C_{S, \mathbb G, p}\frac{\left[ \int _{\mathbb {G}}\int _{\mathbb {G}}\frac{|u(x)-u(y)|^{p}}{\left| y^{-1} x\right| ^{Q+ps}} dxdy+\int _{\mathbb {G}}|u(x)|^{p})dx\right] }{\Vert u\Vert _{L^p(G)}^p} \right) , \end{aligned}$$

where $C_{S, \mathbb G, p}$ is given by

$$\begin{aligned} C_{S, \mathbb G, p}:=\left( \frac{s}{Qd}\right) ^{\frac{sp}{Q}}. \end{aligned}$$

Here $d=I(\phi ):=\inf _{u\in \mathcal {N}}I(u)$, $\phi$ being a least energy solution of (1.3).

### Proof

The logarithmic Hölder inequality (7.2) in the case when $\mathbb {X}=(\mathbb G, dx)$ and the fractional subelliptic Sobolev inequality (1.24) imply that

7.7 $$\begin{aligned} \int _{\mathbb G} \frac{|u(x)|^p}{\Vert u\Vert _{L^p(\mathbb G)}^p}&\log \left( \frac{|u(x)|^p}{\Vert u\Vert ^p_{L^p(\mathbb G)}} \right) dx \le \frac{q}{q-p}\log \left( \frac{\Vert u\Vert ^p_{L^q(\mathbb G)}}{\Vert u\Vert _{L^p(\mathbb G)}^p} \right) \nonumber \\&\le \frac{q}{q-p}\log \left( C_{S, \mathbb G} \frac{\left[ \int _{\mathbb {G}}\int _{\mathbb {G}}\frac{|u(x)-u(y)|^{p}}{\left| y^{-1} x\right| ^{Q+ps}} dxdy+\int _{\mathbb {G}}|u(x)|^{p})dx\right] }{\Vert u\Vert _{L^p(\mathbb G)}^p} \right) . \end{aligned}$$

Observe that the only place where the parameter *q* appears in (7.7) are constants $\frac{q}{q-p}$ and $C_{S, \mathbb G}.$ Therefore, by minimizing the constant $\frac{q}{q-p}$ over the range of $q \in (p, p_s^*)$ we get that $\underset{{q \in (p, p_s^*)}}{\min } \frac{q}{q-p}= \frac{\frac{Qp}{Q-ps}}{\frac{Qp}{Q-ps}-p}= \frac{Q}{sp}.$ Therefore, from (7.7) we deduce that

$$\begin{aligned} \int _{\mathbb G} \frac{|u(x)|^p}{\Vert u\Vert _{L^p(\mathbb G)}^p} \le \frac{Q}{sp}\log \left( C_{S, \mathbb G, p} \frac{\left[ \int _{\mathbb {G}}\int _{\mathbb {G}}\frac{|u(x)-u(y)|^{p}}{\left| y^{-1} x\right| ^{Q+ps}} dxdy+\int _{\mathbb {G}}|u(x)|^{p})dx\right] }{\Vert u\Vert _{L^p(\mathbb G)}^p} \right) , \end{aligned}$$

where the constant $C_{S, \mathbb G, p}$ is given by

$$\begin{aligned} C_{S, \mathbb G, p}:=\limsup _{q \rightarrow \frac{Qp}{Q-ps}}C_{S, \mathbb G}=\limsup _{q \rightarrow \frac{Qp}{Q-ps}}\left( \frac{pq}{q-p} d \right) ^{\frac{p-q}{q}}=\left( \frac{s}{Qd}\right) ^{\frac{sp}{Q}}, \end{aligned}$$

completing the proof. $\square$

The following theorem gives the subelliptic fractional logarithmic Sobolev inequality for homogeneous fractional Sobolev spaces on stratified Lie groups.

### Theorem 7.4

Let $s \in (0, 1),$ $p \in (1, \infty ), Q>ps,$ and $p<q<p^*:=\frac{Qp}{Q-ps},$ where *Q* is the homogeneous dimension of a stratified Lie group $\mathbb {G}.$ Then for any $u \ne 0$, we have

7.8 $$\begin{aligned} \int _{\mathbb G} \frac{|u(x)|^p}{\Vert u\Vert _{L^p(\mathbb G)}^p} \log \left( \frac{|u(x)|^p}{\Vert u\Vert ^p_{L^p(\mathbb G)}} \right) dx \le \frac{Q}{s} \log \left( C_{GN, \mathbb G}^{\frac{ps}{Q(q-p)}}\frac{\left( \int _{\mathbb {G}}\int _{\mathbb {G}}\frac{|u(x)-u(y)|^{p}}{\left| y^{-1} x\right| ^{Q+ps}} dxdy\right) ^{\frac{1}{p}}}{\Vert u\Vert _{L^p(G)}} \right) , \end{aligned}$$

where $C_{GN, \mathbb G}$ is the best constant in the fractional subelliptic Gagliardo-Nirenberg inequality given by

$$\begin{aligned} C_{GN, \mathbb G}:=\frac{pqs}{pqs-Q(q-p)}\left( \frac{Q(q-p)}{pqs-Q(q-p)}\right) ^{\frac{Q(p-q)}{sp^2}}\left( \frac{pqs-Q(q-p)}{(q-p)s} d\right) ^{\frac{p-q}{p}}. \end{aligned}$$

Here $d=I(\phi ):=\inf _{u\in \mathcal {N}}I(u)$, $\phi$ being a least energy solution of (1.3).

### Proof

Using the fractional subelliptic Gagliardo-Nirenberg inequality (1.19) and the logarithmic Hölder inequality (7.5), we obtain that

$$\begin{aligned}&\int _{\mathbb G} \frac{|u(x)|^p}{\Vert u\Vert _{L^p(\mathbb G)}^p} \log \left( \frac{|u(x)|^p}{\Vert u\Vert ^p_{L^p(\mathbb G)}} \right) dx \le \frac{q}{q-p}\log \left( \frac{\Vert u\Vert ^p_{L^q(\mathbb G)}}{\Vert u\Vert _{L^p(\mathbb G)}^p} \right) \\  &\quad \le \frac{q}{q-p} \log \left( C_{GN, \mathbb G}^{\frac{p}{q}}\frac{\left( \int _{\mathbb {G}}\int _{\mathbb {G}}\frac{|u(x)-u(y)|^{p}}{\left| y^{-1} x\right| ^{Q+ps}} dxdy\right) ^{\frac{Q(q-p)}{spq}}\left( \int _{ \mathbb {G}}|u|^{p} dx\right) ^{\frac{spq-Q(q-p)}{spq}}}{\Vert u\Vert ^p_{L^p(G)}} \right) \\  &\quad =\frac{q}{q-p} \log \left( C_{GN, \mathbb G}^{\frac{p}{q}}\frac{\left( \int _{\mathbb {G}}\int _{\mathbb {G}}\frac{|u(x)-u(y)|^{p}}{\left| y^{-1} x\right| ^{Q+ps}} dxdy\right) ^{\frac{Q(q-p)}{spq}}}{\Vert u\Vert ^{\frac{Q(q-p)}{sq}}_{L^p(G)}} \right) \\&\quad = \frac{Q}{s} \log \left( C_{GN, \mathbb G}^{\frac{ps}{Q(q-p)}}\frac{\left( \int _{\mathbb {G}}\int _{\mathbb {G}}\frac{|u(x)-u(y)|^{p}}{\left| y^{-1} x\right| ^{Q+ps}} dxdy\right) ^{\frac{1}{p}}}{\Vert u\Vert _{L^p(G)}} \right) , \end{aligned}$$

completing the proof of (7.8). $\square$

## Data Availability

Data sharing is not applicable to this article as no datasets were generated or analyzed during the current study.

## References

[1] Adimurthi, A., Mallick, A.: A Hardy type inequality on fractional order Sobolev spaces on the Heisenberg group. *Ann. Sc. Norm. Super. Pisa Cl. Sci. (5)*, **18**(3), 917–949, (2018)

[2] Aubin, T.: Problèmes isoperimétriques et espaces de Sobolev. J. Diff. Geom. **11**(4), 573–598 (1976)

[3] Bahouri, H., Fermanian-Kammerer, C., Gallagher, I.: Dispersive estimates for the Schrödinger operator on step-2 stratified Lie groups. Anal. PDE **9**(3), 545–574 (2016)

[4] Beckner, W.: Inequalities in Fourier analysis. Ann. Math. **102**(1), 159–182 (1975)

[5] Beckner, W.: Geometric asymptotics and the logarithmmic Sobolev inequality. Forum Math. **11**(1), 105–137 (1999)

[6] Beckner, W.: Pitt’s inequality and the fractional Laplacian: sharp error estimates. Forum Math. **24**(1), 177–209 (2012)

[7] Bellazzini, J., Frank, R.L., Visciglia, N.: Maximizers for Gagliardo-Nirenberg inequalities and related non-local problems. Math. Ann. **360**(3–4), 653–673 (2014)

[8] Bonfiglioli, A., Lanconelli, E., Uguzzoni, F.: Stratified Lie Groups and Potential Theory for their Sub-Laplacians. Springer, Berlin Heidelberg (2007)

[9] Bhimani, D.G., Hajaiej, H., Haque, S., Luo, T.: A sharp Gagliardo-Nirenberg inequality and its application to fractional problems with inhomogeneous nonlinearity. Evol. Equ. Control Theory **12**(1), 362–390 (2023)

[10] Bonforte, M., Grillo, G.: Direct and reverse Gagliardo-Nirenberg inequalities from logarithmic Sobolev inequalities. Bull. Pol. Acad. Sci. Math. **53**(3), 323–336 (2005)

[11] Bonforte, M., Dolbeault, J., Nazaret, B., Simonov, N.: Stability in Gagliardo–Nirenberg-Sobolev Inequalities Flows, Regularity, and the Entropy method. Mem. Amer. Math. Soc., 308, Article No. 1554, viii+166 (2025)

[12] Branson, T.H., Fontana, L., Morpurgo, C.: Moser-Trudinger and Beckner-Onofri’s inequalities on the CR sphere. Ann. of Math. (2), **177**(1) 1–52 (2013)

[13] Brezis, H.: Functional Analysis, Sobolev Spaces and Partial Differential Equations. Springer, New York, NY, xiv+599 (2011)

[14] Brezis, H., Browder, F.: Partial differential equations in the 20th century. Adv. Math. **135**(1), 76–144 (1998)

[15] Brezis, H., Lieb, E.: A relation between pointwise convergence of functions and convergence of functionals. Proceedings of the American Mathematical Society **88**(3), 486–490 (1983)

[16] Brasco, L., Mosconi, S.J.N., Squassina, M.: Optimal decay of extremals for the fractional Sobolev inequality. *Calc. Var. Partial Differential Equations* 55(2) 32 Art. 23, (2016)

[17] Chatzakou, M., Kassymov, A., Ruzhansky, M.: Logarithmic Sobolev-type inequalities on Lie groups. J. Geom. Anal., **34**(9), 275,40 (2024)

[18] Chatzakou, M., Ruzhansky, M.: Revised logarithmic Sobolev inequalities of fractional order. Bull. Sci. Math. 197, 103530, 9 (2024)

[19] Chatzakou, M., Kassymov, A., Ruzhansky, M.: Logarithmic Sobolev, Hardy and Poincaré inequalities on the Heisenberg group. (2023). arXiv:2310.00992

[20] Capogna, L., Danielli, D., Garofalo, N.: An embedding theorem and the Harnack inequality for nonlinear subelliptic equations. Comm. Partial Differential Equations **18**(9–10), 1765–1794 (1993)

[21] Cotsiolis, A., Tavoularis, N.K.: Best constants for Sobolev inequalities for higher order fractional derivatives. J. Math. Anal. Appl. **295**(1), 225–236 (2004)

[22] Cartan, E.: Sur la représentation géométrique des systémes matériels non holonomes. * Proc. Internat. Congress Math.*, Bologna,**4** 253–261, (1928)

[23] Ciatti, P., Cowling, M.G., Ricci, F.: Hardy and uncertainty inequalities on stratified Lie groups. Adv. Math. **277**, 365–387 (2015)

[24] Chen, L., Lu, G., Zhu, M.: Least energy solutions to quasilinear subelliptic equations with constant and degenerate potentials on the Heisenberg group. Proc. Lond. Math. Soc. (3) **126**(2) 518–555, (2023)

[25] Chen, J., Rocha, E.M.: A class of sub-elliptic equations on the Heisenberg group and related interpolation inequalities. In: Advances in Harmonic Analysis and Operator Theory. Volume 229 of Operator Theory: Advances and Applications, pp. 123–137. Birkhäuser/Springer Basel AG, Basel (2013)

[26] Christodoulou, D.: On the geometry and dynamics of crystalline continua. Ann. Inst. H. Poincaré Phys. Théor. **69**(3), 335–358 (1998)

[27] Citti, G., Manfredini, M., Sarti, A.: Neuronal oscillations in the visual cortex: -convergence to the Riemannian Mumford-Shah functional. SIAM J. Math. Anal. **35**(6), 1394–1419 (2004)

[28] Dao, A.N., Lam, N., Lu, G.: Gagliardo-Nirenberg type inequalities on Lorentz, Marcinkiewicz and weak- spaces. Proc. Amer. Math. Soc. **150**(7), 2889–2900 (2022)

[29] Danielli, D., Garofalo, N., Nhieu, D.M.: Sub-Riemannian calculus on hypersurfaces in Carnot groups. Adv. Math. **215**(1), 292–378 (2007)

[30] del Pino, M., Dolbeault, J.: Best constants for Gagliardo-Nirenberg inequalities and applications to nonlinear diffusions. J. Math. Pures Appl. **81**(9), 847–875 (2002)

[31] Del Pino, M., Dolbeault, J.: Nonlinear diffusions and optimal constants in Sobolev type inequalities: asymptotic behaviour of equations involving the -Laplacian. C. R. Math. Acad. Sci. Paris **334**(5), 365–370 (2002)

[32] Del Pino, M., Dolbeault, J.: Asymptotic behavior of nonlinear diffusions. Math. Res. Lett. **10**(4), 551–557 (2003)

[33] Del Pino, M., Dolbeault, J.: The optimal euclidean -Sobolev Logarithmic inequality. J. Funct. Anal. **197**(1), 151–161 (2003)

[34] Dolbeault, J.: Sobolev and Hardy-Littlewood-Sobolev inequalities: duality and fast diffusion. Math. Res. Lett. **18**(6), 1037–1050 (2011)

[35] Dolbeault, J., Esteban, M.J., Laptev, A., Loss, M.: One-dimensional Gagliardo-Nirenberg-Sobolev inequalities: remarks on duality and flows. J. Lond. Math. Soc. **90**(2), 525–550 (2014)

[36] Dolbeault, J., Toscani, G.: Stability results for logarithmic Sobolev and Gagliardo-Nirenberg inequalities. Int. Math. Res. Not. IMRN **2016**(2), 473–498 (2016)

[37] Esfahani, A.: Anisotropic Gagliardo-Nirenberg inequality with fractional derivatives. Z. Angew. Math. Phys. **66**(6), 3345–3356 (2015)

[38] Ferrari, F., Franchi, B.: Harnack inequality for fractional sublaplacians in Carnot groups. Math. Z. **279**(1), 435–458 (2015)

[39] Ferrara, M., Bisci, G.M., Repov, D.: Nonlinear elliptic equations on Carnot groups. RACSAM, **111**(3) 707–718 (2017)

[40] Fila, M., Winkler, M.: A Gagliardo-Nirenberg-type inequality and its applications to decay estimates for solutions of a degenerate parabolic equation. Adv. Math. 357, 106823, 40 (2019)

[41] Fischer, V., Ruzhansky, M.: Quantization on Nilpotent Lie Groups. Progress in Mathematics, Springer International Publishing, Birkhäuser, **314** (2016)

[42] Folland, G.B.: Subelliptic estimates and function spaces on nilpotent Lie groups. Ark. Mat. **13**(1–2), 161–207 (1975)

[43] Folland, G.B.: On the Rothschild-Stein lifting theorem. Comm. Partial Differential Equations **2**(2), 165–191 (1977)

[44] Folland, G.B., Stein, E.M.: Hardy Spaces on Homogeneous Groups, Mathematical Notes, vol. 28. Princeton University Press, Princeton, N.J. (1982)

[45] Frank, R.L., del Mar Gonzàlez, M., Monticelli, D.D., Tan, J.: An extension problem for the CR fractional Laplacian. Adv. Math. **270**, 97–137 (2015)

[46] Frank, R.L., Lieb, E.H.: Sharp constants in several inequalities on the Heisenberg group. Ann. of Math. (2), **176**(1) 349–381, (2012)

[47] Gentil, I.: The general optimal -Euclidean Logarithmic Sobolev Inequality by Hamilton-Jacobi equations. J. Funct. Anal. **202**(2), 591–599 (2003)

[48] Gilbarg, D., Trudinger, N.S.: Elliptic Partial Differential Equations of Second Order. GMW 224, Springer Verlag, Heidelberg, (1977)

[49] Gromov, M.: Carnot-Carathéodory Spaces Seen from Within. Sub-Riemannian Geometry. Progr. Math., Birkhäuser, Basel, **144**, 79–323, (1996)

[50] Gagliardo, E.: Ulteriori proprietà di alcune classi di funzioni in più variabili. Ricerche mat. **8**, 24–51 (1959)

[51] Garofalo, N., Lanconelli, E.: Existence and nonexistence results for semilinear equations on the Heisenberg group. Indiana Univ. Math. J. **41**(1), 71–98 (1992)

[52] Garofalo, N., Loiudice, A., Vassilev, D.: Optimal decay for solutions of nonlocal semilinear equations with critical exponent in homogeneous group. Proc. Roy. Soc. Edinburgh Sect. A. 29 (2024). First View. 10.1017/prm.2024.58

[53] Gassot, L.: Radially symmetric traveling waves for the Schrödinger equation on the Heisenberg group. Pure Appl. Anal. **2**(4), 739–794 (2020)

[54] Gassot, L.: On the orbital stability of a family of travelling waves for the cubic Schrödinger equation on the Heisenberg group. Bull. Soc. Math. France **149**(1), 15–54 (2021)

[55] Ghosh, S., Kumar, V., Ruzhansky, M.: Compact embeddings, eigenvalue problems, and subelliptic Brezis-Nirenberg equations involving singularity on stratified Lie groups. Math. Ann. **388**(4), 4201–4249 (2024)

[56] Hajaiej, H., Molinet, L., Ozawa, T., Wang, B.: Necessary and sufficient conditions for the fractional Gagliardo-Nirenberg inequalities and applications to Navier-Stokes and generalized boson equations. Harmonic analysis and nonlinear partial differential equations. 159–175, RIMS Kôkyûroku Bessatsu, B26, Res. Inst. Math. Sci. (RIMS), Kyoto, (2011)

[57] Hajaiej, H., Yub, X., Zhai, Z.: Fractional Gagliardo-Nirenberg and Hardy inequalities under Lorentz norms. J. Math. Anal. Appl. **396**(2), 569–577 (2012)

[58] Ivanov, S.P., Vassilev, D.N.: Extremals for the Sobolev inequality and the quaternionic contact Yamabe problem. World Sci. Publ., xviii+219 Hackensack, NJ, (2011)

[59] Jerison, D.S., Lee, J.M.: Extremals of the Sobolev inequality on the Heisenberg group and the CR Yamabe problem. J. Amer. Math. Soc. **1**(1), 1–13 (1988)

[60] Kristály, A.: Nodal solutions for the fractional Yamabe problem on Heisenberg groups. Proc. Roy. Soc. Edinburgh Sect. A. **150**(2), 771–788 (2020)

[61] Kassymov, A., Ruzhansky, M., Suragan, D.: Fractional logarithmic inequalities and blow-up results with logarithmic nonlinearity on homogeneous groups. NoDEA Nonlinear Differential Equations Appl. **27**(1), 7 (2020)

[62] Kassymov, A., Ruzhansky, M., Suragan, D.: Fractional Gagliardo-Nirenberg, weighted Caffarelli-Kohn-Nirenberg and Lyapunov-type inequalities, and applications to Riesz potentials and -sub-Laplacian systems. Potential Anal. **59**(4), 1971–1994 (2023)

[63] Kassymov, A., Suragan, D.: Lyapunov-type inequalities for the fractional -sub-Laplacian. Adv. Oper. Theory **5**(2), 435–452 (2020)

[64] Lions, P.-L.: The concentration-compactness principle in the calculus of variations. The locally compact case II. Ann. Inst. H. Poincaré Anal. Non Linéaire, 1(4) 223–283, (1984)

[65] Lions, P.-L.: The concentration-compactness principle in the calculus of variations. The locally compact case I. Ann. Inst. H. Poincaré Anal. Non Linéaire, **1**(2) 109–145 (1984)

[66] Manfredini, M., Palatucci, G., Piccinini, M., Polidoro, S.: Hölder continuity and boundedness estimates for nonlinear fractional equations in the Heisenberg group. J. Geom. Anal., 33(3), 77, 41 (2023)

[67] Loiudice, A.: Critical growth problems with singular nonlinearities on Carnot groups. Nonlinear Anal. **126**, 415–436 (2015)

[68] Loiudice, A.: Optimal decay of -Sobolev extremals on Carnot groups. J. Math. Anal. and Appl. **470**(1), 619–631 (2019)

[69] Maz’ya, V., Shaposhnikova, T.: On the Brezis and Mironescu conjecture concerning a Gagliardo-Nirenberg inequality for fractional Sobolev norms. J. Math. Pures Appl. (9), **81**(9) 877–884, (2002)

[70] Merker, J.: Generalizations of logarithmic Sobolev inequalities. Discrete Contin. Dyn. Syst. Ser. S **1**(2), 329–338 (2008)

[71] Morosi, C., Pizzocchero, L.: On the constants for some fractional Gagliardo-Nirenberg and Sobolev inequalities. Expo. Math. **36**(1), 32–77 (2018)

[72] Nezza, E.D., Palatucci, G., Valdinoci, E.: Hitchhiker’s guide to the fractional Sobolev spaces. Bull. Sci. Math. **136**(5), 521–573 (2012)

[73] Nirenberg, L.: On elliptic partial differential equations. Ann. Scuola Norm. Sup. Pisa **3**(13), 115–162 (1959)

[74] Palatucci, G., Piccinini, M.: Nonlocal Harnack inequalities in the Heisenberg group. Calc. Var. Partial Differential Equations, **61**(5), 185, 30 (2022)

[75] Pucci, P., Temperini, L.: Existence for singular critical exponential () equations in the Heisenberg group. Adv. Calc. Var. **15**(3), 601–617 (2022)

[76] Rothschild, L.P., Stein, E.M.: Hypoelliptic differential operators and nilpotent groups. Acta Math. **137**(3–4), 247–320 (1976)

[77] Rothschild, L.P.: A remark on hypoellipticity of homogeneous invariant differential operators on nilpotent Lie groups. Comm. Partial Differential Equations **8**(15), 1679–1682 (1983)

[78] Ruzhansky, M., Suragan, D.: Hardy inequalities on homogeneous groups: 100 years of Hardy inequalities. Progress in Mathematics, xvi+588 pp., Birkhaüser, **327** (2019)

[79] Ruzhansky, M., Tokmagambetov, N., Yessirkegenov, N.: Best constants in Sobolev and Gagliardo-Nirenberg inequalities on graded groups and ground states for higher order nonlinear subelliptic equations. Calc. Var. Partial Differential Equations, 59(5), 175, 23 (2020)

[80] Rodemich, E.: The Sobolev inequalities with best possible constants. Analysis seminar at California Institute of Technology, (1966)

[81] Roncal, L., Thangavelu, S.: Hardy’s inequality for fractional powers of the sub-Laplacian on the Heisenberg group. Adv. Math. **302**, 106–158 (2016)

[82] Roncal, L., Thangavelu, S.: An extension problem and trace Hardy inequality for the sub-Laplacian on H-type groups. Int. Math. Res. Not. IMRN **2020**(14), 4238–4294 (2020)

[83] Stein, E.M.: Some problems in harmonic analysis suggested by symmetric spaces and semi-simple groups, Actes du Congrés International des Mathématiciens (Nice, 1970), Tome 1, 173–189. Gauthier-Villars, Paris (1971)

[84] Szökefalvi-Nagy, B.: Über Integralungleichungen zwischen einer Funktion und ihrer Ableitung (German). Acta Univ. Szeged. Sect. Sci. Math. **10**, 64–74 (1941)

[85] Villani, C.: Optimal transport. Grundlehren der mathematischen Wissenschaften, 338, Springer, Berlin, (2009)

[86] Talenti, G.: Best constant in Sobolev inequality. Annali di Matematica **110**(1), 353–372 (1976)

[87] Wang, Y., Yang, Q.: Sharp fractional Sobolev and related inequalities on -type groups. arXiv preprint, (2024). arXiv:2406.16278

[88] Wang, S.: Lieb’s and Lions’ type theorems on Heisenberg group and applications. Math. Methods Appl. Sci. **46**(6), 6743–6755 (2023)

[89] Weinstein, M.I.: Nonlinear Schrödinger equations and sharp interpolation estimates. Comm. Math. Phys., 87(4):567–576, 1982/1983

[90] Zhang, Y.: Optimizers of the Sobolev and Gagliardo-Nirenberg inequalities in . Calc. Var. Partial Differential Equations, **60**(1), 10, 24 (2021) 10.1007/s00526-025-03191-3 PMC12701863 41399752

